# The *Dictyostelium* Model for Mucolipidosis Type IV

**DOI:** 10.3389/fcell.2022.741967

**Published:** 2022-04-13

**Authors:** Claire Y. Allan, Paul R. Fisher

**Affiliations:** Department of Physiology, Anatomy and Microbiology, La Trobe University, Melbourne, VIC, Australia

**Keywords:** mucolipidosis IV, calcium signalling, acidic vesicles, lysosomes, endocytosis, plasma membrane, aggregation, growth

## Abstract

Mucolipidosis type IV, a devastating neurological lysosomal disease linked to mutations in the transient receptor potential channel mucolipin 1, TRPML1, a calcium permeable channel in the membranes of vesicles in endolysosomal system. TRPML1 function is still being elucidated and a better understanding of the molecular pathogenesis of Mucolipidosis type IV, may facilitate development of potential treatments. We have created a model to study mucolipin function in the eukaryotic slime mould *Dictyostelium discoideum* by altering expression of its single mucolipin homologue, *mcln*. We show that in *Dictyostelium* mucolipin overexpression contributes significantly to global chemotactic calcium responses in vegetative and differentiated cells. Knockdown of mucolipin also enhances calcium responses in vegetative cells but does not affect responses in 6–7 h developed cells, suggesting that in developed cells mucolipin may help regulate local calcium signals rather than global calcium waves. We found that both knocking down and overexpressing mucolipin often, but not always, presented the same phenotypes. Altering mucolipin expression levels caused an accumulation or increased acidification of Lysosensor Blue stained vesicles in vegetative cells. Nutrient uptake by phagocytosis and macropinocytosis were increased but growth rates were not, suggesting defects in catabolism. Both increasing and decreasing mucolipin expression caused the formation of smaller slugs and larger numbers of fruiting bodies during multicellular development, suggesting that mucolipin is involved in initiation of aggregation centers. The fruiting bodies that formed from these smaller aggregates had proportionately larger basal discs and thickened stalks, consistent with a regulatory role for mucolipin-dependent Ca^2+^ signalling in the autophagic cell death pathways involved in stalk and basal disk differentiation in *Dictyostelium*. Thus, we have provided evidence that mucolipin contributes to chemotactic calcium signalling and that *Dictyostelium* is a useful model to study the molecular mechanisms involved in the cytopathogenesis of Mucolipidosis type IV.

## 1 Introduction

Transient receptor potential mucolipins (TRPML) belong to a conserved family of ion channels (Dong et al., 2010), which localize to the membranes of components of the endocytic pathway. The three mammalian mucolipin homologues, TRPML1, TRPML2 and TRPML3, are encoded by the genes *MCOLN 1–3* (Cheng et al., 2010; Grimm et al., 2012). TRPML channels are non-selective cation channels permeable to a range of cations. Alongside two pore channels (TPC), TRPML channels are believed to facilitate endolysosomal Ca^2+^ signalling which regulates trafficking and sorting of the membranes of endocytic vesicles (Cheng et al., 2010), organelle homeostasis, compartmental acidification (Gerasimenko et al., 1998; Dong et al., 2010; Morgan et al., 2011) and homotypic and heterotypic vesicle fusion and reformation (Pryor et al., 2000; Hay, 2007; Luzio et al., 2007; Brailoiu and Brailoiu, 2016; Cao et al., 2017). Additionally, TRPML1 is implicated in regulation of autophagy, mechanistic target of rapamycin (mTOR) and transcription factor EB (TFEB) signalling (Sun et al., 2018; Boudewyn and Walkley, 2019). Loss of function mutations in TRPML1, causes the neurological disease Mucolipidosis Type IV (MLIV) (Sun et al., 2000), and TRPML-associated dysfunction in the endolysosomal system is implicated in other neurodegenerative diseases (Santoni et al., 2020; Lee et al., 2021). Therefore, TRPMLs may underpin the pathophysiology of neurodegenerative disease more generally and are suggested to be a therapeutic target.

It is not well understood how loss TRPML1 function causes lysosomal storage and neuronal dysfunction, but it is believed that Ca^2+^ signalling plays a central role. The vesicles of the endolysosomal system are essential to maintenance of cellular homeostasis through macromolecule recycling and are important Ca^2+^ storage organelles which are integral to cellular Ca^2+^ signalling (Patel and Docampo, 2010; Patel and Cai, 2015) and can help regulate ER calcium signals by sequestering calcium (López-Sanjurjo et al., 2013; López-Sanjurjo et al., 2014). Ca^2+^-dependent signalling is thought to regulate the soluble N-ethylmaleimide-sensitive-factor attachment protein receptors (SNARE) complex proteins, which are proteins involved in the fusion of vesicle membranes (Bharat et al., 2014; Han et al., 2017). Alongside SNAREs, the protein early endosome antigen-1 (EEA1) is necessary for vesicle tethering prior to fusion, and is regulated by Ca^2+^/calmodulin dependent signalling through its IQ domain (Mills et al., 1998; Christoforidis et al., 1999; Lawe et al., 2003). TRPML specific perilysosomal calcium signals have been notoriously difficult to measure due to the localised nature of the signals which are often clouded by larger global calcium signals. In an attempt to resolve the local calcium signals genetically encoded calcium sensors directly tagged to TRPML1 have been used to measure local calcium transients (Shen et al., 2012; Cao et al., 2015; Medina et al., 2015). The TRPML agonist ML-SA1 stimulates calcium release into the cytoplasm (Shen et al., 2012), which in some cell types is independent of extracellular and ER calcium pools (Gómez et al., 2018). However, some evidence shows that TRPML channels can affect ER calcium release and activate influx of calcium across the plasma membrane to contribute to global calcium signals (Kilpatrick et al., 2016), and small calcium release from the acidic stores can prime and amplify calcium release from the ER (Ronco et al., 2015).

A variety of model organisms have been used to study TRPML proteins including the mouse (Kim et al., 2007; Micsenyi et al., 2009; Curcio-Morelli et al., 2010), zebrafish (Benini et al., 2013), *Drosophila* (Venkatachalam et al., 2008; Wong et al., 2012; Feng et al., 2014), *C. elegans* (Fares and Greenwald, 2001; Treusch et al., 2004) and yeast (Denis and Cyert, 2002). One important cellular model, the eukaryotic social amoeba, *Dictyostelium*, encodes a single mucolipin homologue, *mcln* (Wilczynska et al., 2005; Lima et al., 2012) which has been the subject of only a limited number of studies. *Dictyostelium* is a well-established model to study neurodegenerative and lysosomal disease (Annesley and Fisher 2009; Maniak, 2011; Annesley et al., 2014; Martín-González et al., 2021). This model organism has been extensively employed to study neuronal ceroid lipofuscinosis (NCL) (Huber, 2020), a group of lysosomal diseases linked to mutations in the genes, *CLN1–CLN8* and *CLN10–CLN14* (Yap et al., 2021). Therefore, *Dictyostelium* is an ideal model to study lysosomal disorders and mucolipin function. In other work, expression of FLAG-tagged mucolipin was used to show that the protein localizes predominately to post-lysosomes, but may also be present in other endocytic compartments (Lima et al., 2012), and accordingly controls lysosome exocytosis. Evidence suggests that mucolipin is involved in Ca^2+^ homeostasis and signalling because knockout cells had reduced Ca^2+^ concentrations in secretory lysosomes (Lima et al., 2012) and are also defective in rheotaxis, a calcium-regulated mechanosensing mechanism (Lima et al., 2014). The role of this putative channel in Ca^2+^ signalling has not been studied, so it was of particular interest to investigate the role of mucolipin in chemotactic calcium signalling in *Dictyostelium*.

*Dictyostelium* is a well-established model to study calcium signalling, and cells experience cytosolic calcium transients when stimulated with various extracellular stimuli including the chemoattractants cAMP and folic acid (Nebl and Fisher, 1997; Traynor et al., 2000; Nebl et al., 2002; Schaloske et al., 2005; Fisher and Wilczynska, 2006; Malchow et al., 2008). These Ca^2+^ signals originate from the extracellular environment, the endoplasmic reticulum (ER) (Wilczynska et al., 2005) and the contractile vacuole (CV) (Malchow et al., 2006; Gross, 2009). Other intracellular Ca^2+^ stores including mitochondria, acidocalcisomes and vesicles of the endocytic pathway are likely to also contribute to the calcium responses. Given the localisation of mucolipin at the post-lysosomes and potentially other vesicles of the endocytic pathway, we decided to determine if mucolipin and these endocytic vesicles are involved in cytosolic Ca^2+^ responses to chemoattractants.

To achieve this, we created mucolipin overexpression and knockdown strains which coexpress the calcium-sensitive luminescent protein apoaequorin which allowed us to analyze real-time chemotactic induced cytoplasmic Ca^2+^ responses (Nebl and Fisher, 1997). Our experiments revealed that when overexpressed, mucolipin is involved in chemoattractant-elicited Ca^2+^ responses because overexpression strains had enhanced responses to both cAMP and folic acid. Surprisingly, chemoattractant Ca^2+^ responses in knockdown strains were also enhanced, but only in vegetative cells. Responses in aggregation-competent cells were not affected in the knockdown strains, suggesting that, unless overexpressed, mucolipin is not a major contributor to cAMP-mediated calcium responses. However, it may be involved in local Ca^2+^ signalling associated with changes in the vesicle trafficking pathways throughout the developmental cycle, in particular aggregation center formation and autophagic cell death. In other work, the cellular phenotypes of growth, endocytosis and multicellular development were found to be unaffected in mucolipin knockout cells created from the parental strain DH1-10 (Lima et al., 2012). Therefore, we decided to analyze these phenotypes in our strains which were created from the parental strain AX2. We discovered that mucolipin expression did affect growth rates and nutrient uptake via macropinocytosis and phagocytosis in AX2. Surprisingly however, the phenotypes were not linear in that both increasing and decreasing expression of mucolipin often, but not always, resulted in the same phenotypic outcome. Both overexpression and knockdown strains had increased fluorescence in cells stained with Lysosensor blue, indicating either increased acidification of the vesicles or increased abundance of the vesicles. Phagocytosis and pinocytosis rates were upregulated, but this did not correlate with an increase in growth rates, which suggests defects in catabolism, in endocytic vesicle trafficking or in endolysosomal breakdown of macromolecular contents. Mucolipin is involved in the regulation of aggregation as both overexpression and knockdown strains formed smaller, more numerous slugs and fruiting bodies than AX2, a phenotype that is also present in other *Dictyostelium* lysosomal disease models of NCL (Huber et al., 2014; Huber et al., 2017; Smith et al., 2019; McLaren et al., 2021). Increased numbers of cells entering the autophagic cell death pathway were also evident in mucolipin mutants because they had thickened stalks compared to AX2. Our results suggest that normal growth and development of *Dictyostelium* is sensitive to mucolipin expression, and our strains show similar phenotypes to other *Dictyostelium* lysosomal disease models further affirming that *Dictyostelium* is an ideal model to study lysosomal disease.

## 2 Materials and Methods

### 2.1 Gene Cloning and Sequence Analysis

To create a mucolipin overexpression construct, the 2,599 bp *mcln* genomic DNA (DictyBase gene no. DDB_G0291275) was amplified in two sections from parental strain AX2 genomic DNA using the primers MUF (CGC​GGA​TCCATC​GAT *ATG*ACA​TCT​TTT​AAA​GGT​GAC​AG) and MuMR (AAC​TAA​C**GGT​ACC**AGG​TAC​TTC) for the 5′ fragment, and MuMF (GAA​GTA​CCT**GGT​ACC**GTT​AGT​TC) and MUR2 (CGCGGA​TCCCTC​GAG​CAT​CAT​ATC TCAATACCTGAATC) for the 3′ fragment. A three fragment ligation allowed the full length genomic DNA to be cloned into the bacterial vector pZErO™-2(Invitrogen, Carlsbad, CA, United States) with the restriction enzyme *Bam*HI (underlined). To join the 5′ and 3′ fragments of the gene together, the primers used in amplification of the two fragments spanned a region of the gene that encodes the restriction site *Kpn*I (bold) this allowed seamless ligation of the two gene fragments. The full-length gene was then subcloned for overexpression into the *Dictyostelium* vector pA15GFP using the restriction cut sites *Cla*I and *Xho*I, this plasmid was named pPROF638. To create the *mcln* antisense RNA inhibition plasmid (pPROF650) a fragment encoding 1411bp of the gene from position 1365bp to position 3776bp in the gDNA sequence was amplified via PCR with the primers MuMF (GAA​GTA​CCT**GGT​ACC**GTT​AGT​TC) and MUR2 (CGCGGA​TCCCTC​GAG​CAT​CAT​ATC TCAATACCTGAATC) from genomic DNA extracted from vegetative AX2 cells using DNAzol^®^ (Invitrogen). The products were then cloned into pZErO™-2 using *Bam*H1 and *Kpn*1 and subcloned into the *Dictyostelium* expression vector pDNeo2 (Witke et al., 1987) with *Bam*H1 and *Kpn*1. Clones were verified by restriction digestion as well as sequencing at the Australian Genome Research Facility, Brisbane, Australia. Sequence analyses, alignments, and database searches were conducted using Web-based software through DictyBase.org (Fey et al., 2009), ExPASy and the Australian Genome Research Facility.

### 2.2 Strains and Culture Conditions

All experiments were conducted with *Dictyostelium* parental strain AX2, and transformants derived from it (names beginning with HPF). Each transformant strain carried multiple copies of the constructs: 1) pPROF120 (apoaequorin expression plasmid) and pPROF650 (antisense strains: HPF812-818, HPF640); 2) pPROF120 and pPROF652 (sense strains: HFP819 and HPF861) and 3) pPROF120 and pPROF638 (overexpression strains: HPF820-829, HFP654-656). The Ca(PO_4_)_2_/DNA coprecipitation method was used to isolate all transformants, by cotransformation with both plasmids (Nellen et al., 1984). Transformants were selected as isolated colonies on lawns of *Micrococcus luteus* (Wilczynska and Fisher, 1994) on standard medium agar [(SM) 1.0% (w/v) Oxoid agar; 1.0% (w/v) Oxoid bacteriological peptone; 1.0% (w/v) glucose; 0.1% (w/v) Oxoid yeast extract; 4.1 mM MgSO_4_·7H_2_O; 16.2 mM KH_2_PO_4_; 5.8 mM K_2_HPO_4_ supplemented with 20 μg ml^−1^ Geneticin (G-418) (Promega Corporation, Madison, WI, United States)]. *D. discoideum* cells were grown on lawns of *Enterobacter aerogenes* as a food source on SM (Standard Medium) nutrient agar and incubated at 21°C for 3–4 days and subcultured as required from a single colony. To prepare liquid cultures, 5–10 spores were inoculated into a well of a 24-well Costar plate containing 1.5 ml HL5 axenic medium (1% (w/v) Bacto™ proteose peptone; 0.5% (w/v) Bacto™ yeast extract; 2.8 mM Na_2_HPO_4_·2H_2_O; 2.6 mM KH_2_PO_4_; 1% (w/v) glucose pH 6.4) supplemented with geneticin (20 μg ml^−1^), ampicillin (100 μg ml^−1^), streptomycin (500 μg ml^−1^) and tetracycline (100 μg ml^−1^), and incubated at 21°C to allow spores to germinate and form amoebae. Once confluence was reached 1 ml of culture was inoculated into 10 ml HL5 in T25 flask (Falcon) and cells were grown to the density of 1–2 × 10^6^ cells ml^−1^ shaking at 150 rpm and 21°C. Strains were then subcultured at least once into HL5 without antibiotics and grown for 24–48 h prior to use in phenotypic assays to remove possible effects of the antibiotics on phenotypic readouts. Unless otherwise stated, cells were harvested by centrifugation at 500 ×*g* for 5 min.

### 2.3 Estimation of Plasmid Copy Number

Genomic DNA was extracted from transformants using DNAzol^®^ (Invitrogen). Quantitative Southern blot was used to estimate plasmid copy number of *mcln* overexpression strains (Fernando et al., 2020). DNA loaded gels were stained with SYBR® Green I nucleic acid gel stain (Sigma-Aldrich®) and DNA was quantified using the Storm 860 Fluroimager (GE Healthcare, United Kingdom) in fluorescence mode. Southern blots of the same DNA samples were quantitated using fluorescein-labelled DNA probes, in conjunction with anti-fluorescein alkaline peroxidase conjugate antibody, and enhanced with the chemi-fluorescein substrate (GE Healthcare, United Kingdom). The Storm 860 Fluroimager fluorescence mode was used to quantitate Southern blots.

Quantitative real time PCR was used to estimate plasmid copy number in *mcln* antisense RNA strains (Fernando et al., 2020). SYBR^®^ Green (BioRad, Hercules, CA, United States) was used for amplicon detection of a 135 bp fragment of *mcln* with the primers (500 nM final concentration) MF1.1 (5-GAT​TGG​TCT​TGG​TAC​TTT​GTT​A-3) and MR1.2 (5-GGG​AGA​CTT​CCA​GCC​GAG-3), within the genomic DNA extracts, and to construct a standard curve, over a range of DNA concentrations using purified pPROF650 as a template. To quantify genomic DNA extracts a 100 bp filamin amplicon was amplified with the primers FIL1588F (5-CCC​TCA​ATG​ATG​AAG​CC-3) and FIL1688R (5-CCA​TCT​AAA​CCT​GGA​CC-3) in genomic DNA from all strains, and the concentrations calculated from a standard curve. The PCR amplification used 35 cycles of denaturation at 95°C for 30 s, annealing at 58°C for 30 s and extension and data collection at 72°C for 1 min.

### 2.4 Expression Analysis

Quantitative real time RT-PCR was used to quantitate expression levels of *mcln* messenger RNA. RNA was collected from 10^7^ vegetative cells or developing cells harvested from water agar at the time points indicated over the course of development, and extracted using TRIzol^®^ (Invitrogen). The iScript™ One-Step RT-PCR Kit with SYBR^®^ Green (BioRad, Hercules, CA, United States) was used for amplicon detection of a 135 bp fragment of *mcln* with the primers MF1.1 and MR1.2, and a 100 bp amplicon of the constitutively expressed protein filamin, with the primers FIL1588F and FIL1688R. A total RNA template of 50–100 ng was added to the total reaction mixture of 50 μl; cDNA synthesis was performed at 50°C for 10 min, and the PCR amplification used 35 cycles of denaturation at 95°C for 30 s, annealing at 58°C for 30 s and extension and data collection at 72°C for 1 min. Expression levels were normalised against the filamin levels to adjust for loading and then measured relative to AX2 controls.

### 2.5 Protein Techniques

#### 2.5.1 Antibody Production

A polyclonal antibody called anti-MUECD directed against the *Dictyostelium* mucolipin was used in the study. A 200aa His-tagged portion of mucolipin (aa101–aa301) was expressed in *E. coli* and purified by Genscript^®^. Polyclonal antibodies directed against MUECD were raised in a rabbit by the Institute of Medical and Veterinary Science (Adelaide, SA, Australia), and affinity purified as described previously (Smith and Fisher, 1984).

#### 2.5.2 Western Blot

Cells were grown in HL5 medium and 1 × 10^6^ cells harvested and lysed in 100 µl 1 × Bolt LDS sample buffer (Thermo Fisher Scientific) with a protease inhibitor 1 cocktail (Complete-EDTA free, Roche) on ice from 30 min then centrifuged at 1,200 rpm for 2 min. 10 µl of lysate was mixed with 1% Bolt sample reducing agent (Thermo Fisher Scientific) was incubated at 70°C for 10 min then loaded onto a Bolt™ 4–12%, Bis-Tris-Plus, 1.0 mm, Protein Gel, 12-well (Invitrogen) with the Broad Multi Color Pre-Stained Protein Standard (Genscript) and subject to electrophoresis. To visualise the protein bands, protein was transferred to PVDF membrane (Amersham Hybond™-P, GE Healthcare) for 1 h at 100 V at 4°C. Protein was visualised on the membrane by staining with No Stain Protein Labelling Reagent (Invitrogen) as per the manufactures instructions, and visualised on a Bio Rad Chemidoc MP imaging system. Membraned we then blocked with 1% casein in 1 × TBS for 3 h RT and probed with polyclonal rabbit anti-MUECD 1:500 in 1% casein in 1 × TBS with 0.1% Tween-20 for 2 h RT. The SNAP i.d.^®^ 2.0 Protein Detection System was used for application of anti-rabbit HRP conjugate (Life technologies) 1: 10,000 in TBS-T as per manufacturer’s instructions. Bands were detected with Clarity Western ECL substrate (Bio Rad), with chemiluminescence imaged on Amersham Imager 600 (GE Healthcare Life Sciences). The intensities of the total loaded protein in each lane and specific antigen bands were quantified digitally using the Image Quant TL 1D v 8.1 software 19 (GE Healthcare Life Sciences).

### 2.6 Determination of Growth Rates

#### 2.6.1 Growth on Bacterial Lawns

Growth rates of *Dictyostelium* on lawns of bacteria were measured as previously described (Bokko et al., 2007). Lawns of *E*. *coli* B2 were prepared on normal agar [20 g L^−1^ agar (Difco, Detroit, MI, United States); 1 g L^−1^ peptone (Oxoid, Basingstoke, United Kingdom), 1.1 g L^−1^ anhydrous glucose, 1.9972 g L^−1^ KH_2_PO_4_, and 0.356 g L^−1^ Na_2_HPO_4_·2H_2_O, pH 6.0]. An aliquot of 20 μl of *Dictyostelium* culture at a density of 1 × 10^6^ cells ml^−1^, was inoculated onto the center of each lawn and incubated at 21°C for 100 h, during which time the plaque diameter (mm) was measured at intervals of 8–12 h. The recorded values were analyzed by linear regression using the “R” environment for statistical computing and graphics (http://www.R-project.org) to determine the plaque expansion rate (mm/h).

#### 2.6.2 Growth Rates of Axenically Growing Cultures

Generation times of axenically growing *Dictyostelium* cultures was measured as previously described (Bokko et al., 2007). Cultures of *Dictyostelium* were grown to exponential phase in HL5 medium to a density of ∼1–2 × 10^6^ cells ml^−1^ and were used to inoculate 50 ml of fresh HL5 medium (no antibiotics) to an initial density of 1 × 10^4^ cells ml^−1^. Cultures were incubated at 21°C on an orbital shaker at 150 rpm for 100 h, during which time cell densities were determined at 8–12 h intervals, by counting 10 μl aliquots using a hemocytometer. The cell densities were then analyzed by log-linear regression using the “R” programming environment computer software to determine the generation time from the exponential growth curve.

### 2.7 Measurements of Macropinocytosis Rates

Measurements of macropinocytosis rates as previously described (Bokko et al., 2007), were performed using fluorescein isothiocyanate (FITC)-dextran (Sigma-Aldrich; average mol. mass, 70 kDa; working concentration, 2 mg ml^−1^ in HL5 growth medium). Cultures of axenically growing *Dictyostelium* cells were harvested, and resuspended in fresh HL5 medium and shaken at 150 rpm for 30 min at 21°C, and after the addition of FITC-dextran, duplicate aliquots at time points 0 and 70 min were lysed in 0.25% (vol/vol) Triton X-100 in 100 mM Na_2_HPO_4_, pH 9.2 and the fluorescence of the lysate was measured in a Modulus fluorometer (Turner BioSystems) using the (Green Module 525 nm excitation and 580–640 nm emission). The cell density, increase in fluorescence over 70 min, and a separate calibration curve relating fluorescence signal to the volume of fluorescent medium, were used to calculate the hourly rate of uptake of medium.

### 2.8 Measurements of Phagocytosis Rates

The rate of uptake of *E. coli* expressing the fluorescent protein DsRed (Maselli et al., 2002) was used to measure phagocytosis rates in *Dictyostelium* strains. DsRed-expressing *E. coli* (DsRed-Ec) cells were harvested from NB cultures (containing 75 μg ml^−1^ ampicillin and 1 mM IPTG), grown at 37°C shaking for 24 h and resuspended to a density of 2–4 × 10^10^ bacteria ml^−1^ in 20 mM Sorenson’s buffer (2.353 mM Na_2_HPO_4_2H_2_O and 17.65 mM KH_2_PO_4_, pH 6.3). The density and fluorescence of the bacterial culture in a given experiment, were used to determine the fluorescence signal per million bacteria. A separate calibration curve was used to determine the relationship between OD600 and the density of the bacterial suspension.

Cultures of *Dictyostelium* were harvested, washed, and resuspended in Sorenson’s buffer at 1 × 10^6^ cells ml^−1^ and starved for 30 min at 21°C shaking. Following the 30 min incubation 1 ml of the prepared DsRed-Ec suspension was added to the *Dictyostelium* cultures and immediately duplicate ^0^T aliquots were removed and added to preprepared ice cold 20 mM Sorenson’s buffer containing 5 mM sodium azide. The remaining amoebae were allowed to uptake DsRed-Ec for 30 min with shaking. During this time amoebae in the ^0^T samples were collected by centrifugation at 1, 000 × g for 30 s and cells washed with 20 mM Sorenson’s buffer containing 5 mM sodium azide, resuspended in 3 ml of 20 mM Sorenson’s buffer and counted using a hemocytometer. After 30 min of uptake duplicate T30 aliquots of the amoebae/DsRed-Ec suspension we removed and added to preprepared ice cold 20 mM Sorenson’s buffer containing 5 mM sodium azide and the amoebae washed free of bacteria. A Modulus fluorometer 9200-003 (Turner Bio-Systems, Sunnyvale, CA, United States) fitted with a specially constructed module designed for DsRed-Ec (530-nm excitation and 580-nm emission) was used to measure fluorescence in samples that had been lysed by the addition of 0.25% (v/v) Triton X-100 in Na_2_HPO_4_. Increase in fluorescence over 30 min was used to calculate ingestion of DsRed-Ec cells per hour by a single amoeba.

ΔF (bacteria ml^−1^) = difference in fluorescence over the course of the assay (fluorescence at T30–T0).

Uptake (bacteria amoeba^−1^ h^−1^) = ΔF × 10^6^/total cell count (amoebae ml^−1^)/time (h).

### 2.9 Quantification of Aggregate Size

Quantification of aggregate size was achieved by determining the number of cells in individual slugs. Strains were grown to exponential phase in HL5 medium and 1 × 10^7^ cells were harvested, washed 2 × in PBS and resuspended in 1 ml 1 × PBS. The suspension was then inoculated onto a water agar plate (10 g agar/1 L dH_2_O) in a 1 cm × 5 cm line, to give a density of 2 × 10^6^ cells/cm^2^ and allowed to dry. The plate was placed in a dark container with a single point of light source and incubated at 21°C until slugs had formed and migrated away from the point of inoculation (typically ∼12–16 h). Individual slugs were then isolated using a sterile needle and resuspended in 100 μl of saline and dissociated by repeat pipetting. The number of cells per slug was then calculated after counting the number of cells in duplicate 10 μl suspensions using a hemocytometer in 30 individual slugs for each strain.

### 2.10 Morphology, Quantification of Number of Fruiting Bodies and Quantification of Sorus Area

Mature fruiting body morphology was observed, after multicellular development on lawns of *Enterobacter* as described previously (Kotsifas et al., 2002). Images were taken after 24 h of development. Photographs were taken using from above or from the side on an excised piece of agar using a Moticam 2300 camera attached to an Olympus SZ61 stereomicroscope. To quantify the number of fruiting bodies images were taken from above at the same magnification for all strains. The number of fruiting bodies in the same area of photograph were counted and the means calculated. Sorus area was quantified from the same photographs of fruiting bodies from above using the ImageJ measurement tool. The area was then converted to volume on the assumption that the sorus is approximately spherical using equation [V = 4/3 * A/sqrt (A/pi)] (A = area) and presented as mm^3^.

### 2.11 Quantification of LysoSensor™ Blue DND-167 Staining in Cells

*Dictyostelium* cells were grown in HL5 medium to a density of 1–3 × 10^6^ cells ml^−1^1 × 10^7^ cells were harvested (1,500 ×*g* for 2 min) and diluted 1:10 in Lo-Flo HL5 either with or without 500 nM LysoSensor™ Blue DND-167 (Invitrogen) and incubated covered in foil at 21°C shaking for 30 min. Cells were washed twice with 1 × PBS and resuspended to a density of 0.5 × 10^6^ cells ml^−1^ in PBS and the fluorescence of a 2 ml sample measured in a Modulus™ 9200-003 fluorometer (Turner BioSystems, Sunnyvale, CA, United States) using the UV module kit 9200-041 (Ex. 365 nm, Em. 410–460 nm). The background fluorescence of the cells that had not been dyed was subtracted from the fluorescence recorded from the Lysosensor Blue stained cells.

### 2.12 LysoSensor Blue DND-167 Imaging of Live Cells

Cells were grown in HL5 medium to a density of 1–3 × 10^6^ cells ml^−1^. An aliquot containing 10^6^ cells were harvested (1,500 ×*g* for 2 min) and resuspended in Lo-Flo HL5 containing 500 nM LysoSensor Blue DND-167 (Invitrogen) and incubated at 21°C shaking for 30 min. Cells were washed 2 times in 1 × PBS, resuspended to a density of 1 × 10^6^ cells ml^−1^ and 10 μl of suspension mounted onto microscope slides. Live cells were viewed through the DAPI filter with an Olympus BX61 fluorescent microscope.

### 2.13 Measurement of Cellular Autofluorescence

Cells were harvested and processed in the same manner as Section 2.11, however omitting the treatment with LysoSensor ™ Blue DND-167. Fluorescent readings of 1 × 10^6^ cells were recorded immediately after two washes in 1 × PBS with the UV module kit 9200-041 (Ex. 365 nm, Em. 410–460 nm).

### 2.14 Calcium Experiments

#### 2.14.1 *Dictyostelium* Culture and Development

Expression of the Ca^2+^ sensitive luminescent protein aequorin, in each *Dictyostelium*, strain was used to measure cytosolic Ca^2+^ concentration. *Dictyostelium* cultures were grown and allowed to develop as described previously (Nebl and Fisher, 1997). Axenic cultures in HL5 medium were grown to a density of 1–2 × 10^6^ cells ml^−1^, and 1 × 10^8^ cells were harvested, washed twice in 20 ml of MES-DB [(MES development buffer) (10 mM MES/NaOH, pH 6.2, 10 mM KCl, 0.25 mM CaCl_2_)], resuspended in MES-DB or HL5 to a final density of 2 × 10^7^ cells ml^−1^ and loaded with 5 μM Coelenterazine-h (Invitrogen). Cultures were incubated at 21°C shaking at 150 rpm for 4 h (vegetative cells) or 7 h (aggregation competent cells).

#### 2.14.2 Aequorin Consumption and *In Vivo* Ca^2+^ Measurements

Aequorin consumption was carried out as previously described (Nebl and Fisher 1997). All measurements were taken inside a Lumitran^®^ model L-3000 photometer (New Brunswick Scientific). During measurements, the cells were stirred at 100 rpm in 20 ml sample vessels and placed in front of a photomultiplier. Luminescence signals were recorded from 5 ml cell suspensions stimulated with 1 μm chemoattractant, either cAMP or folic acid. The signal was captured by a model PCI-20428 multifunction I/O data acquisition board (Intelligent Instruments Pty. Ltd.). The signal was then converted into values of cytosolic calcium concentration on a PC using in-house purpose-designed software (Prof. P. R. Fisher).

### 2.15 Statistical Analysis

Data was analysed in Excel. Means were compared using independent t-tests or One-Way ANOVA, with pairwise comparisons made by the Least Squares Difference method. Significant correlations were determined by Pearson product-moment, Spearman’s rank and Kendall’s rank correlation coefficients.

## 3 Results

### 3.1 Developmental Expression of *mcln* Over 24 h

**FIGURE 1:**
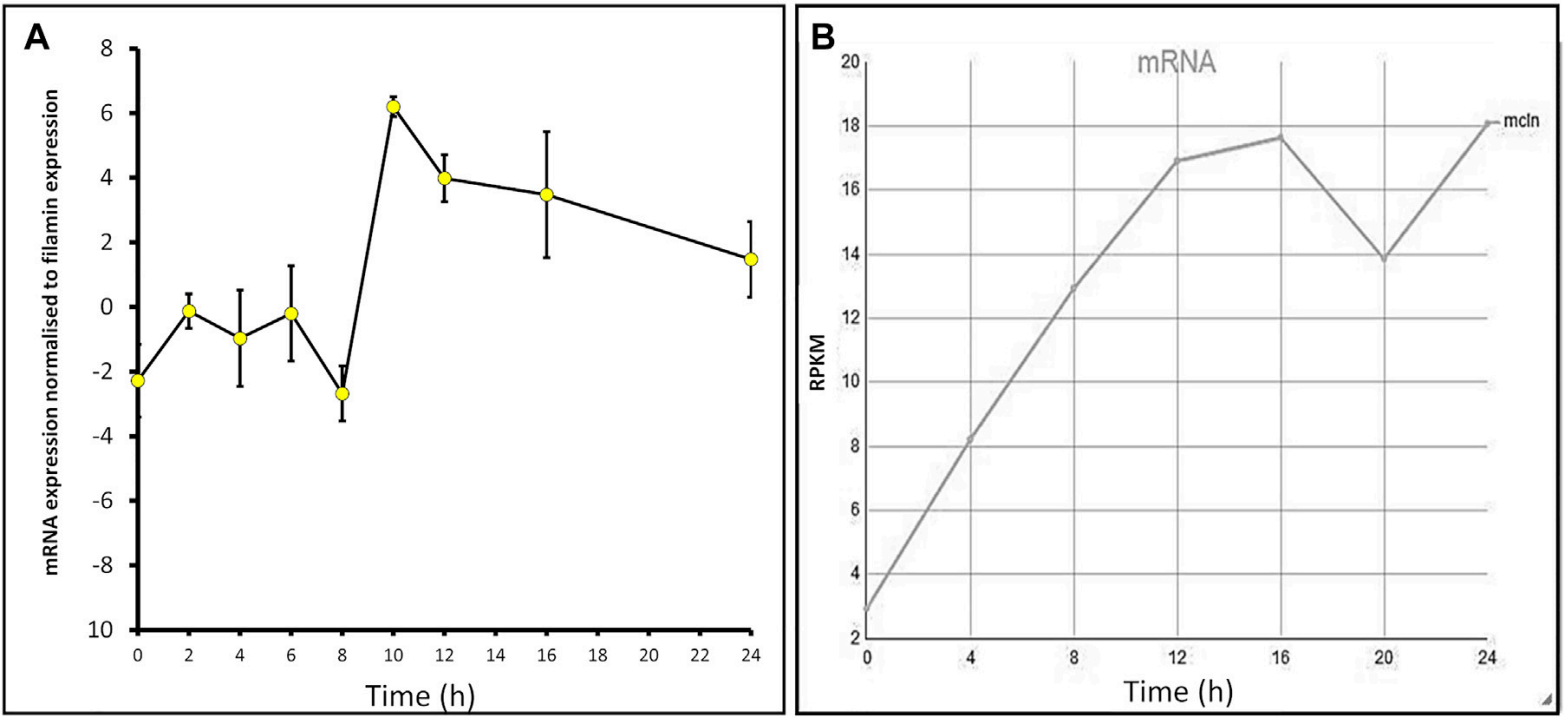
*mcln* mRNA expression increases throughout development in *Dictyostelium* AX2 and AX4. **(A)** Semiquantitative RT-PCR was used to determine the *mcln* expression in AX2 throughout development presented normalised to filamin expression. RNA was extracted from cells developing on water agar every 2 h for 24 h in two separate experiments. Error bars are standard errors of the mean. **(B)** Developmental timing of expression of *mcln* in AX4 as measured by RNA-Seq. Reads per kilobase per million (RPKM) over 24 h. Published on DictyBase (Parikh et al., 2010; Stajdohar et al., 2017).

The expression profiles of many *Dictyostelium* genes change throughout development. To determine the *mcln* mRNA expression levels throughout development, AX2 cells were developed on water agar plates for 24 h, and RNA extracts were taken at 2-h intervals. Semiquantitative RT-PCR was used to assess the relative expression of *mcln* in cells at these time points. Expression changed throughout development, rising slightly during early differentiation in response to the onset of starvation, falling to a trough at 8 h, but then increasing rapidly to a maximum at 10 h during aggregation. Expression remained at high levels during subsequent multicellular development (Figure 1A). The measured developmental expression profile is similar to the RNA-Seq expression profile of *D. discoideum* AX4 published on DictyBase (Parikh et al., 2010; Stajdohar et al., 2017), which shows increased *mcln* expression over the course of development (Figure 1B). Subtle differences in the patterns of expression in our experiments compared to those published on DictyBase (Parikh et al., 2010) could arise from differences in the parental strain used (AX2 in our experiments and AX4 for DictyBase) and also from the fact that in our experiments the cells were developed on water agar, as opposed to filters. This expression pattern suggests important roles for mucolipin-dependent calcium signalling in early starvation-induced differentiation, in chemotactic aggregation and subsequently during the multicellular stages of the life cycle.

### 3.2 Genetic Alteration of *Dictyostelium* Changes *mcln* mRNA Expression

**FIGURE 2:**
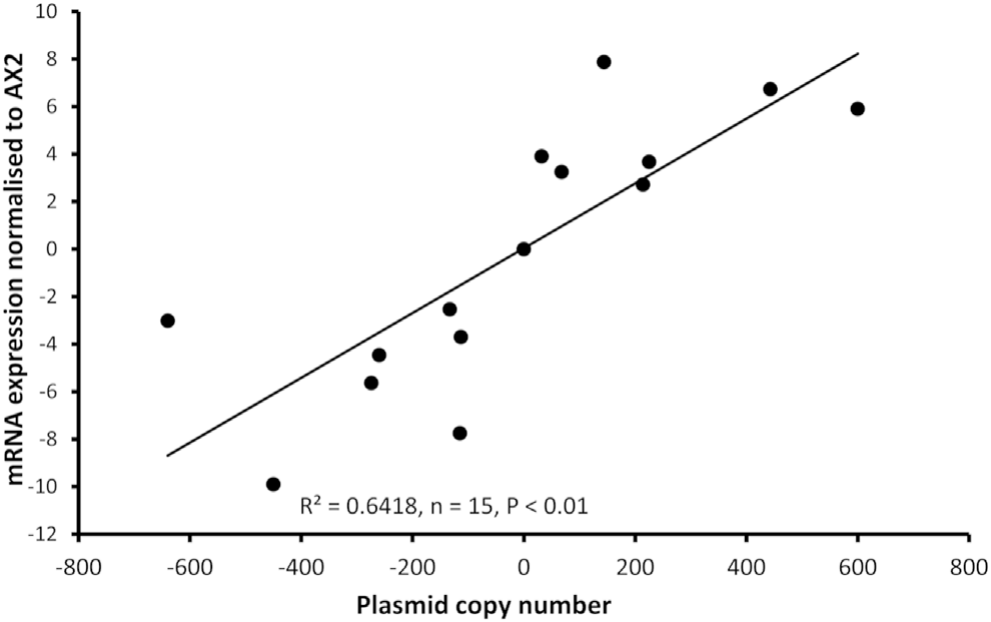
Plasmid copy number of mucolipin strains was correlated with mRNA expression levels in the transformants. Semiquantitative RT-PCR was performed to determine mRNA expression levels in each strain. Copy numbers of overexpressing strains were determined by quantitative Southern blotting, and copy numbers of antisense strains were determined by quantitative real time PCR. Expression levels were normalised against the filamin mRNA to adjust for loading and then measured relative to AX2 expression. Expression correlates with plasmid copy number showing that the antisense RNA construct decreases *mcln* expression and the overexpression increases it. The correlation was highly significant, *p* = 2.36 × 10^−5^ (Pearson product-moment correlation coefficient, ρ) and similar results were also obtained using non-parametric methods (Spearman’s rank *p* = 1.195 × 10^−5^ and Kendall’s rank *p* = 9.995 × 10^−5^). Each point represents mean data for a single strain for three experiments. Negative values refer to copy numbers of antisense constructs and positive values to copy numbers of overexpression constructs, AX2 has a copy number of 0 as contains neither construct.

*Dictyostelium* strains were created by transformation with expression constructs to either knockdown expression via antisense mRNA inhibition or overexpress the protein. Integration of the plasmid construct occurs randomly in the genome by recombination and rolling circle replication, therefore each strain contains different numbers of the plasmid constructs thus different levels of expression (Barth et al., 1998). For this reason, the copy number of the plasmid in each strain was determined, as was the mRNA expression level. As expected, antisense inhibition caused a measurable decrease in mRNA transcripts and overexpression caused an increase in mRNA transcripts. The relative mRNA expression was correlated with the copy number of the plasmid construct in the strains (Figure 2).

### 3.3 Overexpressed Mucolipin Can Be Detected in Western Blot

**FIGURE 3:**
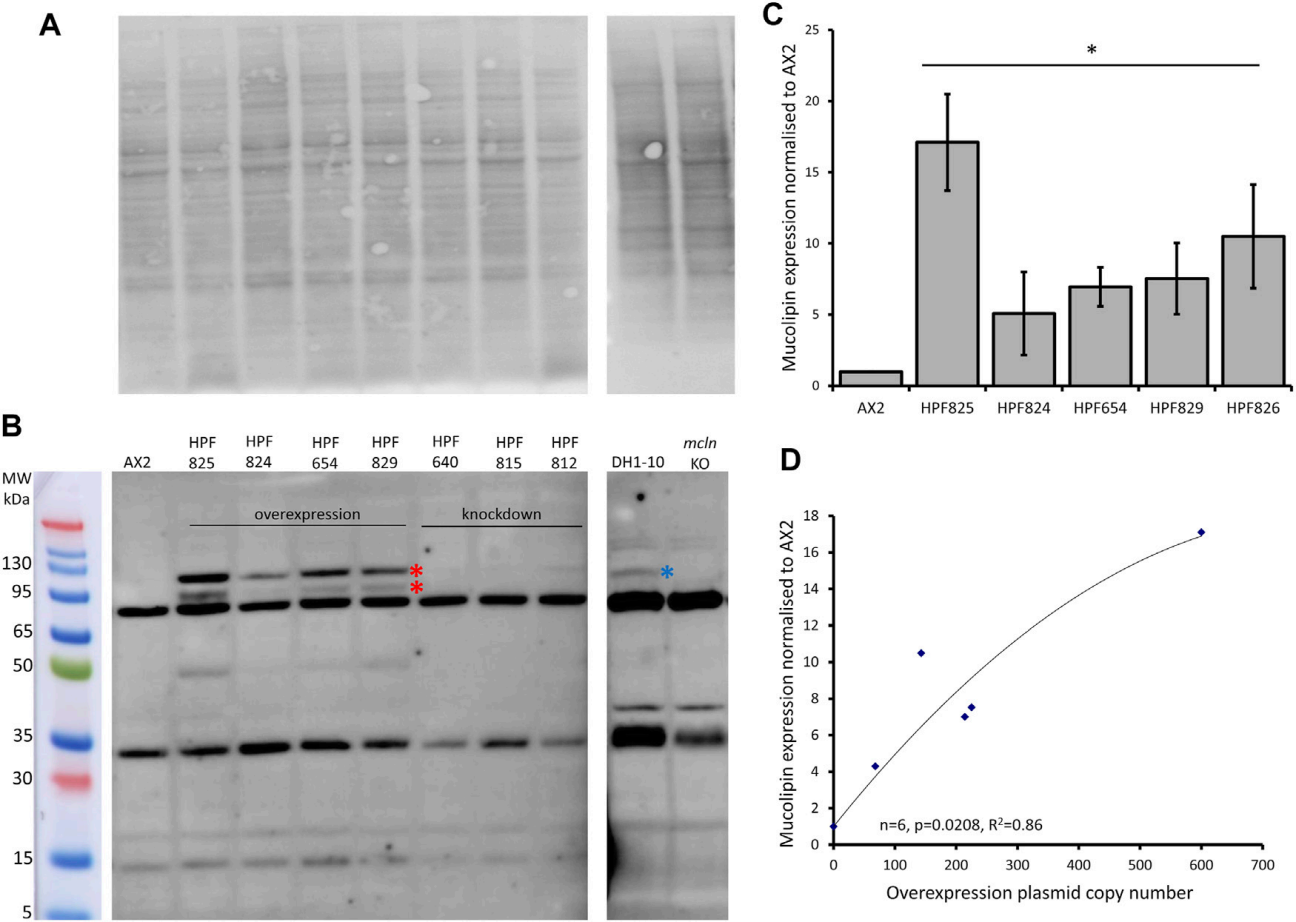
Mucolipin can be detected in protein extracted from overexpression strains. **(A)** Total protein extracted from AX2, four representative mucolipin overexpression strains, three representative mucolipin knockdown strains, DH1-10 and mucolipin knockout, were separated by on a Bis-Tris-Plus gel by electrophoresis, transferred to PVDF membrane and stained with No Stain Protein Labelling Reagent (Invitrogen). Protein lysate for DH1-10 and *mcln* KO were kindly provided by Professor Pierre Cosson (Lima et al., 2012). **(B)** Western blot of the same membrane, the anti-MUECD antibody detects multiple bands. Markers were the Broad Multi Color Pre-Stained Protein Standard (Genscript). Two bands of ∼95 and ∼120 kDa were absent in the mucolipin knockout cells but present in the mucolipin overexpression strains (Red stars) and the higher MW band also detected in DH1-10 (blue star). **(C)** Quantification of mucolipin expression in overexpression strains. To quantify mucolipin expression the combined intensity of the 120 and 95 kDa bands was measured in Image Lab™ and normalised to the intensity of the total protein loaded in each lane **(A)**. Expression in each strain was then and normalised to AX2 expression. As no bands were visualised for AX2 at these sizes, AX2 expression was determined by measuring the intensity of a similar sized area on the blot where the bands would be located. Each strain was tested 2–4 times and data was pooled from each experiment and the means calculated (error bars are standard errors of the mean). Expression is relative to AX2 and is increased in overexpression strains (* = <0.05, One-Way ANOVA, with pairwise comparisons made by the Least Squares Difference method, AX2 vs. strain). **(D)** Protein expression in overexpression strains correlates with overexpression construct copy number. AX2 had a construct copy number of 0 as does not contain an overexpression construct. The correlation was highly significant (Spearman’s rank *p* = 0.0208 and Kendall’s rank *p* = 0.0194).

We created a polyclonal antibody directed against a 200aa portion of *Dictyostelium* mucolipin, anti-MUECD. In a western blot this antibody detected multiple bands so was not suitable to be used for localisation studies. To determine if any of these bands did represent mucolipin, we tested the antibody the against mucolipin knockout cell lysate (Lima et al., 2012) and ran these proteins alongside protein extracted from DH1-10 (the parental strain for the knockout cell line), AX2 and our mucolipin overexpression and knockdown strains. Indeed, there were two bands absent in the mucolipin knockout lysate but present in the mucolipin overexpression strains which ran at approximately 95 and 120 kDa. The 120 kDa band was also detected in DH1-10 (Figures 3A,B). It was not observed in the knockout strain derived from DH1-10, despite a similar protein loading in the gel. These bands were not detected in AX2 or our mucolipin antisense strains, presumably because the wild type and knockdown expression levels for mucolipin were insufficient for detection by our antibody. Mass spectrometry analysis of bands excised from a Coomassie Blue-stained Bis-Tris-Plus gel (Supplementary Figure S1 and Supplementary Method 1) confirmed the presence of mucolipin in these two bands (indicated by the red stars in Figure 3B), but not the smaller molecular weight bands. We thus confirmed that there are two genuine mucolipin bands—one at the predicted MW position ∼95 kDa and one running at ∼120 kDa. There are two possible explanations for the presence of two bands. Firstly, the upper band could be a posttranslationally modified (glycosylated) form of the protein as is reported for human TRPML1 (Miedel et al., 2006), and the lower band could be the native nonglycosylated protein. Secondly, the upper band could be the native, full-length protein running a little more slowly than expected, and the lower band could be a cleavage product. The human TRPML1 has a proteolytic cleavage site in the intralumenal loop between transmembrane domains 1 and 2, and this cleavage is believed to be involved in regulation of channel activity (Kiselyov et al., 2005). Since we confirmed that these bands represented mucolipin, we were able to confirm by quantification that mucolipin expression is increased in our overexpression strains (Figure 3C), and the protein expression was significantly correlated with copy number of the overexpression plasmid construct (Figure 3D).

### 3.4 Mucolipin Contributes to Chemotactic Calcium Signals in *Dictyostelium*

**FIGURE 4:**
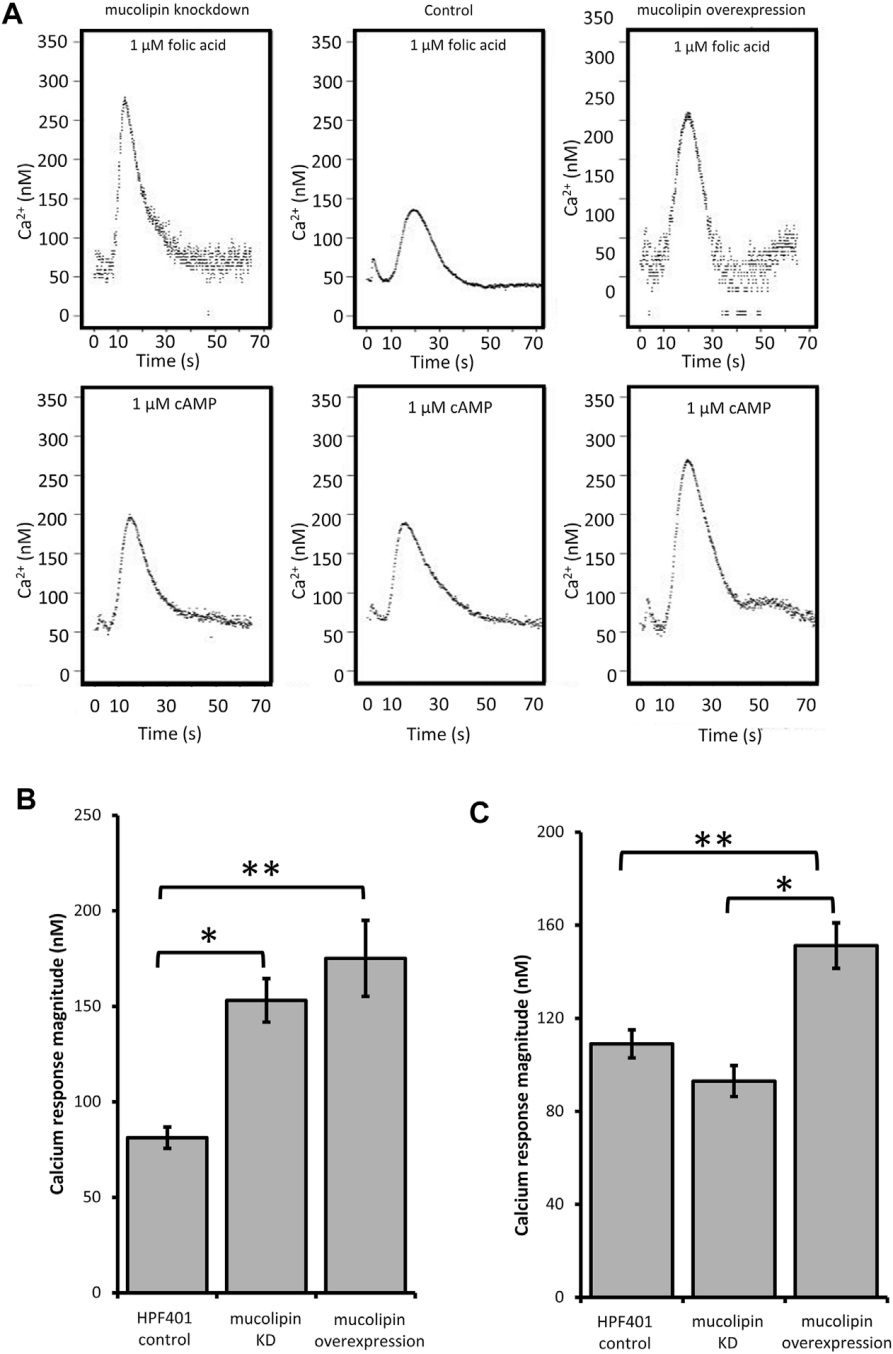
Cytosolic calcium responses to cAMP and folic acid stimulation. **(A)** Representative real time recordings of cytoplasmic Ca^2+^ responses to chemoattractants. Upper panel: Real time recordings of Ca^2+^ responses to 1 μM folate in vegetative cells. Left to right: mucolipin KD (knockdown), HPF401 control strain and mucolipin overexpression. Lower panel: Real time recordings of Ca^2+^ responses to 1 μM cAMP in cells at 7 h development. Left to right: as above. Recordings began at 0 s and the stimulus was injected immediately after. The small peak preceding the Ca^2+^ response is due to a Ca^2+^ response to the mechanical stimulus of chemoattractant injection, which has no effect on the chemoattractant responses (Nebl and Fisher, 1997). **(B)** Mean calcium response magnitudes in vegetative cells stimulation with 1 µM folic acid. Ca^2+^ response magnitudes (Ca^2+^ nM) were measured in mucolipin KD, mucolipin overexpression and control strain HPF401. There was no correlation with copy number so data from strain groups was pooled and the means compared. Both knockdown and overexpression of mucolipin significantly enhances the magnitude of the response compared to the control strain HPF401 (*p = 3.15 × 10^–5^, **p = 4.7 × 10^–4^). Means were compared using an independent one-tailed *t*-test. Errors are standard errors of the mean. Individual strains were tested in 3–9 independent experiments in five mucolipin overexpression (*n* = 13), six mucolipin knockdown strains (*n* = 30) and control strain HPF401 (*n* = 12), and all experiments from each strain group were pooled to determine the mean. **(C)** Mean calcium response magnitudes (Ca^2+^ nM) in developed cells stimulated with 1 µM cAMP. Ca^2+^ response magnitudes were measured in overexpression, knockdown and control strain HPF401. Cells were developed to 7 h in MED-DB. Overexpression of mucolipin increases the response magnitude significantly compared to both control HPF401 and knockdown strains (*p = 1.33 × 10^–6^, **p = 5,928 × 10^–4^). knockdown of mucolipin has no effect on response magnitude in developed cells when compared to wildtype (*p* = 0.083). Means were compared using an independent one-tailed *t*-test. Errors are standard errors of the mean. Individual strains were tested in 2–9 independent experiments and all experiments were pooled to determine the mean. Mucolipin overexpression eight strains (*n* = 37), mucolipin knockdown seven strains (*n* = 33), control HPF401 (*n* = 12).

TRPMLs are thought to be important for Ca^2+^ signalling events associated with Ca^2+^-dependent fusion/fission events of membranes along the late endocytic pathway (LaPlante et al., 2004; Luzio et al., 2007; Brailoiu and Brailoiu 2016). In *Dictyostelium* DH1-10 cells, knockout of mucolipin reduces Ca^2+^ concentrations in secretory post-lysosomes, measured using dextran-coupled fluorophores (Lima et al., 2012) suggesting a role in regulation of calcium homeostasis/signalling. To further investigate the role of mucolipin in Ca^2+^ signalling, and the possible contribution of the acidic stores to chemotactic Ca^2+^ responses, we measured the cytosolic Ca^2+^ transients in vegetative cells (folic acid responses), and in aggregation competent cells (cAMP responses) in mucolipin knockdown and overexpressing strains. All strains also ectopically expressed a recombinant Ca^2+^-sensitive luminescent protein, apoaequorin, to allow real time recordings of cytosolic Ca^2+^ responses. These chemotactic Ca^2+^ responses have been well characterized in wild type cells expressing apoaequorin (HPF401) (Nebl and Fisher, 1997). In this work we compared the mutants’ Ca^2+^ responses with wild type responses to gain insight into the molecular mechanisms involved. Representative real-time recordings of cytosolic calcium concentration in wild type aequorin-expressing strain (HPF401), mucolipin knockdown strain (HPF812) and mucolipin overexpression strain (HPF825) when stimulated with 1 μM chemoattractant are shown in Figure 4A.

#### 3.4.1 Overexpression of Mucolipin Enhances the Ca^2+^ Responses to cAMP and Folic Acid

Real time recordings of the Ca^2+^ responses for control, knockdown and overexpression strains to both cAMP and folic acid were recorded over multiple experiments and analysed. As there was no correlation between the various parameters and plasmid copy number, data was pooled for all transformants within each group for combined analysis. Overexpression of mucolipin significantly enhanced Ca^2+^ response magnitudes to both folic acid (vegetative cells) and cAMP (aggregation competent cells) compared to the control (Figures 4B,C). The response magnitudes were increased compared to the control by an average of 46.4% and 28.0% for the folic acid responses and cAMP responses respectively. This result suggests, mucolipin is involved in Ca^2+^ chemotactic calcium responses and because the channel localises to post-lysosomes and possibly other endocytic compartments (Lima et al., 2012), the results suggest that these vesicles are also involved in the calcium response.

#### 3.4.2 Knockdown of Mucolipin Increases Ca^2+^ Response Magnitudes in Vegetative Cells, but Not in Aggregation Competent Cells

Given that overexpression of mucolipin enhances the responses to folic acid and cAMP, it was expected that knockdown of mucolipin would reduce the magnitude of calcium responses. Surprisingly, the magnitude of the Ca^2+^ responses to 1 µM folic acid was significantly increased in knockdown strains compared to the control (Figure 4B). Contrary to the folate responses, the magnitude of the cAMP Ca^2+^ responses in aggregation competent cells were slightly reduced in mucolipin knockdown strains compared to the control (Figure 4C), but the difference did not reach statistical significance (*p* = 0.083).

### 3.5 The Role of Mucolipin in the Endocytic Pathway

*Dictyostelium* mucolipin overexpression and knockdown strains both exhibit increased Ca^2+^ signalling in vegetative cells. As Ca^2+^ plays an important role in the endocytic pathways it was of interest to assess the role of mucolipin in these processes.

#### 3.5.1 Overexpression and Knockdown of Mucolipin Increases Fluorescence of Cells Stained With LysoSensor™ Blue DND-167

**FIGURE 5:**
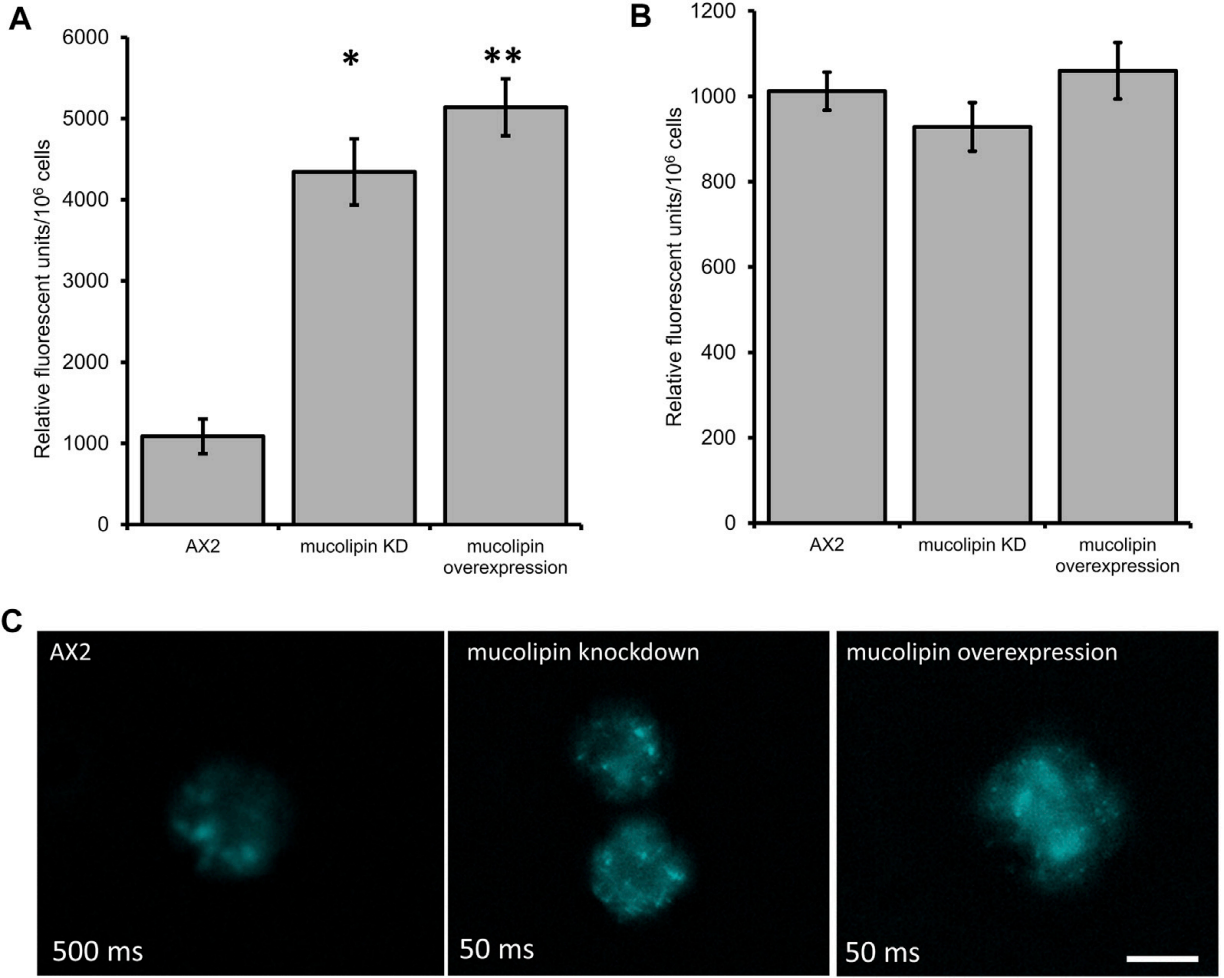
Altering mucolipin expression causes accumulation or increased acidification of vesicles stained with LysoSensor^TM^ Blue DND-167 but does not cause autofluorescence. **(A)** Quantification of LysoSensor™ Blue DND-167 was performed by measuring the increase in fluorescence in 1 × 10^6^ vegetative cells after incubation with 500 nM LysoSensor™ Blue DND-167. Data was collected from seven knockdown strains and six overexpression strains in three individual experiments and the mean calculated, error bars are standard errors of the mean. There was no correlation with copy number of the plasmid constructs, therefore data for strain groups was pooled. The mean fluorescence of AX2 (*n* = 3) was significantly different to the means of either knockdown (*n* = 21) or overexpression strains (*n* = 18) (Pairwise comparisons, Independent *t*-test, *p = 0.00067, **p = 0.01107312). **(B)** Fluorescence measured in *Dictyostelium* strains harvested from low fluorescence medium. The level of fluorescence in knockdown and overexpression strains was measured in 1 × 10^6^ cells. Autofluorescence was measured in a fluorometer (Modulus 9200-003 Turner Bio systems) using the UV module (excitation 365 nm, emission 410–460 nm). Fluorescence displayed in relative fluorescent units, was measured in two independent experiments for AX2 (*n* = 2), six knockdown strains (*n* = 12) and six overexpression strains (*n* = 12) and the mean within each group calculated. Error bars are standard errors of the mean. There was no significant difference (*p* > 0.05, One-Way ANOVA, multiple comparisons made by the Least Squares Difference method). **(C)** Representative live cells stained with LysoSensor™ Blue DND-167. *Dictyostelium* AX2, mucolipin knockdown (copy number 274) and overexpression (copy number 443) were incubated in the presence of 500 nM LysoSensor™ Blue DND-167 for 1 h and observed under fluorescence microscopy (Excitation- 373 nm, Emission- 425 nm). The AX2 image was taken at 500 ms exposure, the knockdown and overexpression cells were overexposed at this exposure so were taken at 50 ms. Scale bar = 5 µm.

One of the hallmarks of MLIV cells is an accumulation of hybrid late endosome-lysosome compartments (LELs) with defective exit of lipids from LELs to the trans-Golgi network (Chen et al., 1998; LaPlante et al., 2004). The connection between Ca^2+^ release via TRPMLs and build-up of hybrid LELs is somewhat undecided. To assess this phenotype in AX2 *Dictyostelium* cells, we stained vegetative mucolipin knockdown and overexpressing cells with LysoSensor™ Blue DND-167 which has a pK_a_ of is ∼5.1 and accumulates in acidic vesicles. There was a significant increase in fluorescence in the mutants compared to AX2 (Figure 5A) representative images of live stained cells are presented in Figure 5C. This could reflect an increase in lysosomal mass, or because the fluorescence of LysoSensor™ Blue DND-167 increases as the pH decreases, our results could reflect increased acidification of the lysosomes.

#### 3.5.2 Altering Mucolipin Expression Does Not Cause Autofluorescence

Accumulation of autofluorescent material has been observed in MLIV cell lines and is thought to be related to specific compounds stored in the lysosomes (Goldin et al., 1995). Autofluorescence is also a hallmark of NCL (Dowson et al., 1982), and has been detected in some *Dictyostelium* NCL models. In the cln3 model, *cln3* ^−^ cells do not accumulate autofluorescent material during the growth stage (Huber et al., 2014), however in starved *cln3* ^−^ cells autofluorescent material was detected (Huber and Mathavarajah, 2019). In the cln2 lysosomal disease model, mutants with knockdown and knockout of the *tpp1/cln2* gene, which encodes the lysosomal protein tripeptidyl peptidase I (TPP-I), a soluble lysosomal aminopeptidase, accumulate cellular autofluorescent material (Phillips and Gomer, 2015; Smith et al., 2019). Therefore, we measured autofluorescence in *Dictyostelium* mucolipin knockdown and overexpression strains under UV light. No increase of autofluorescence was detected in any of our mucolipin mutants (Figure 5B). This phenotype in *Dictyostelium* lysosomal disease models seems to be specific to the particular disease gene in question.

#### 3.5.3 Knockdown of Mucolipin Increases Macropinocytic Uptake, but Decreases Growth Rates of Cells in Liquid Medium, While Overexpression Does Not Affect Growth or Macropinocytosis

**FIGURE 6:**
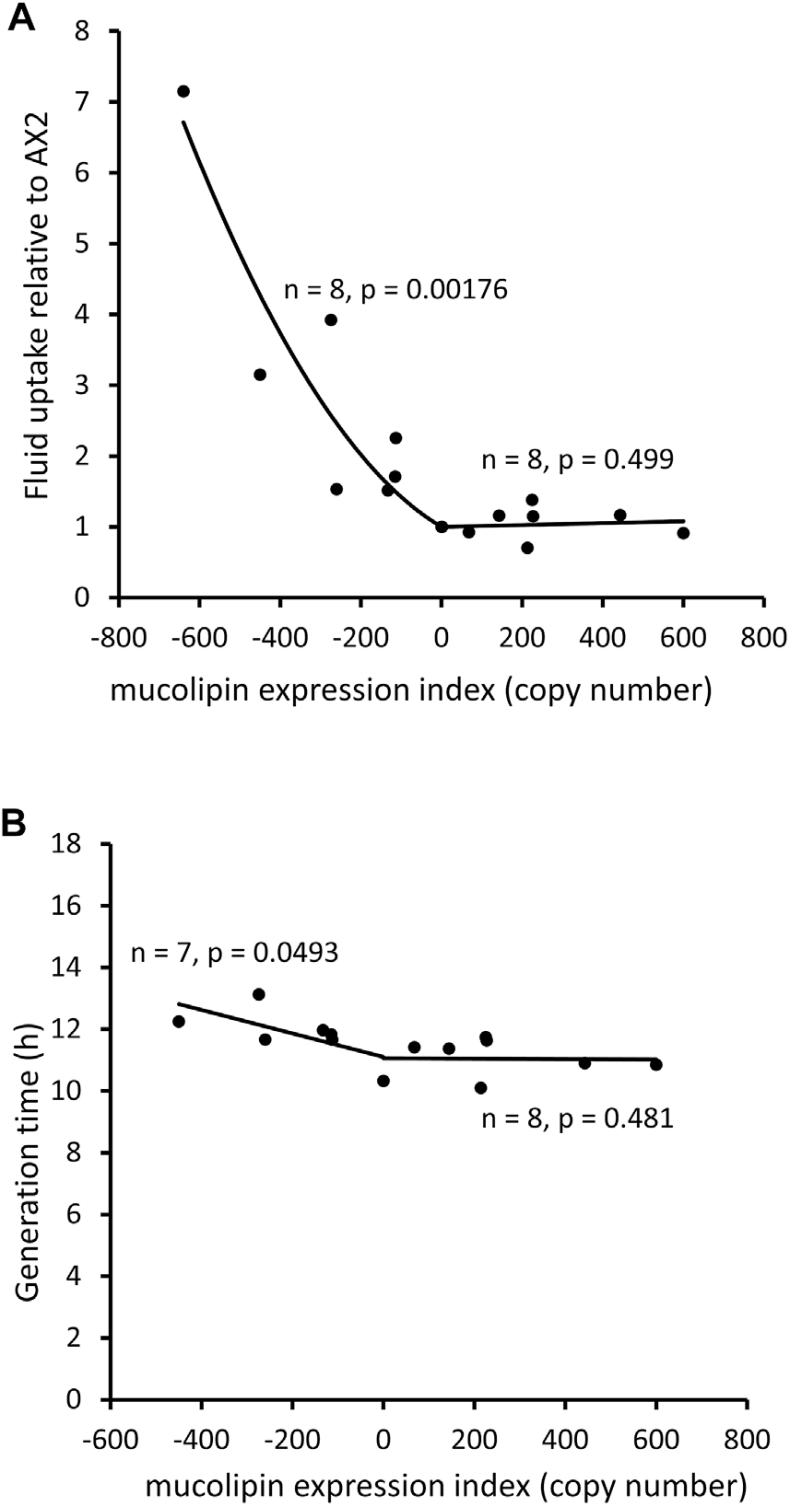
Mucolipin knockdown affects macropinocytic uptake and growth rates of cells in HL5 medium. **(A)** Consumption of HL-5 medium containing FITC dextran via macropinocytosis in knockdown strains (negative copy numbers), overexpression strains (positive copy numbers) and wild type normalised to AX2. In the antisense strains fluid uptake normalized to AX2 was increased and correlates with mucolipin expression index (Pearson correlation coefficient, *p* = 0.00176), however there was no correlation in the overexpression strains (Pearson correlation coefficient, *p* = 0.499). Each point represents the mean uptake from a single strain. Duplicate samples were assayed for each strain in two to six separate experiments and data was normalised within each experiment to AX2. **(B)** Generation times (h) of mucolipin strains and AX2 grown in HL5 medium. In the antisense strains generation time was increased and correlates with mucolipin expression index (Pearson correlation coefficient, *p* = 0.0493), however there was no correlation in the overexpression strains (Pearson correlation coefficient, *p* = 0.481). AX2 has a copy number of 0.

An analysis of uptake of medium as compared to growth rates can indicate whether ingested nutrients are efficiently catabolised. The rate of fluid uptake by macropinocytosis was increased in mucolipin knockdown strains and correlated with plasmid copy number, however overexpression had no affect (Figure 6A). Surprisingly the growth rates of mucolipin knockdown strains were slower than AX2 (longer generation times) and similarly correlated with plasmid copy number, while overexpression had no affect (Figure 6B).

#### 3.5.4 Mucolipin Knockdown and Overexpression Increase Phagocytosis, but Do Not Affect Growth Rates on Lawns of Bacteria

**FIGURE 7:**
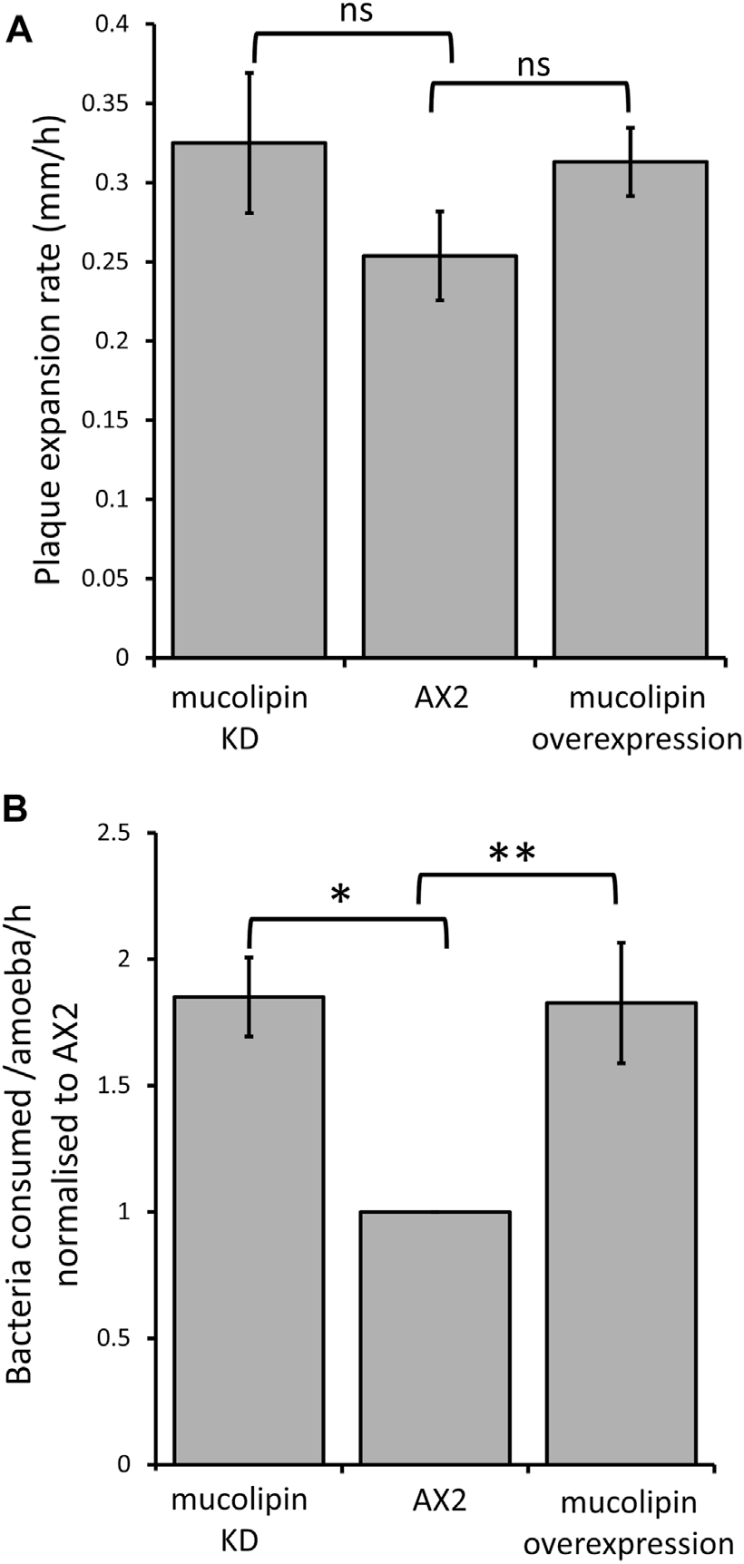
Mucolipin expression did not affect plaque expansion rates but increased phagocytosis rates. **(A)** Plaque expansion rates (growth velocity) were measured from linear regressions of plaque diameters vs. time during growth at 21°C on an *E. coli* B lawn. Growth velocity was not significantly altered in mucolipin knockdown compared to AX2 (*p* = 0.278) or overexpression compared to AX2 (*p* = 0.133) (One-Way ANOVA, with pairwise comparisons made by the Least Squares Difference method). Analysis revealed that all there was no correlation between construct copy number and growth velocity of the mucolipin overexpressing strains therefore, the growth velocities for different strains in each of the two sets of transformants were pooled to calculate the means for comparison with AX2. Individual strains were each tested in 2–4 independent experiments and the data pooled to calculate the mean. Eight overexpression strains (*n* = 33), seven knockdown strains (*n* = 20) and the control strain (*n* = 4) were tested (*n* = total number of experiments in each strain group). Errors are standard errors of the mean. **(B)** Uptake of *E. coli* cells expressing the fluorescent red protein Ds-Red via phagocytosis was significantly increased in both knockdown and overexpression mucolipin transformants when compared to AX2 (*p = 3.35 × 10^–5^, **p = 0.002, One-Way ANOVA, with pairwise comparisons made by the Least Squares Difference method). All of the mucolipin overexpressing strains exhibited similarly elevated phagocytosis rates so that there was no correlation amongst them between construct copy number and phagocytosis rates (Pearson product-moment correlation coefficient, ρ, overexpression *p* = 0.15). The same was true of the mucolipin knockdown strains (Pearson product-moment correlation coefficient, ρ, *p* = 0.48). Accordingly, the uptake rates obtained from different strains within each group were pooled and the means calculated. Individual strains were tested in 2–4 independent experiments. Seven overexpression (*n* = 24), seven knockdown strains (*n* = 19) and the control strain (*n* = 4) were tested (*n* = total number of experiments). Errors are standard errors of the mean.

*Dictyostelium* consume bacteria by phagocytosis in their natural environment and can be cultured on lawns of bacteria where they grow as plaques which gradually expand as the amoebae consume bacteria. The plaque expansion rates (growth velocity) of mucolipin transformants were slightly but not significantly elevated compared to AX2 (Figure 7A). The rates of phagocytosis in mucolipin transformants and AX2 control were assayed by measuring the uptake of fluorescently labelled live *E. coli* cells. Surprisingly, unlike the plaque expansion rates on bacterial lawns, both overexpression and knockdown of mucolipin significantly increased the rates of phagocytosis of *E.coli* (Figure 7B). These results indicate although these transformants are engulfing bacteria at a faster rate than AX2, this does not significantly increase the growth rates.

### 3.6 Mucolipin Expression Affects *Dictyostelium* Multicellular Development

**FIGURE 8:**
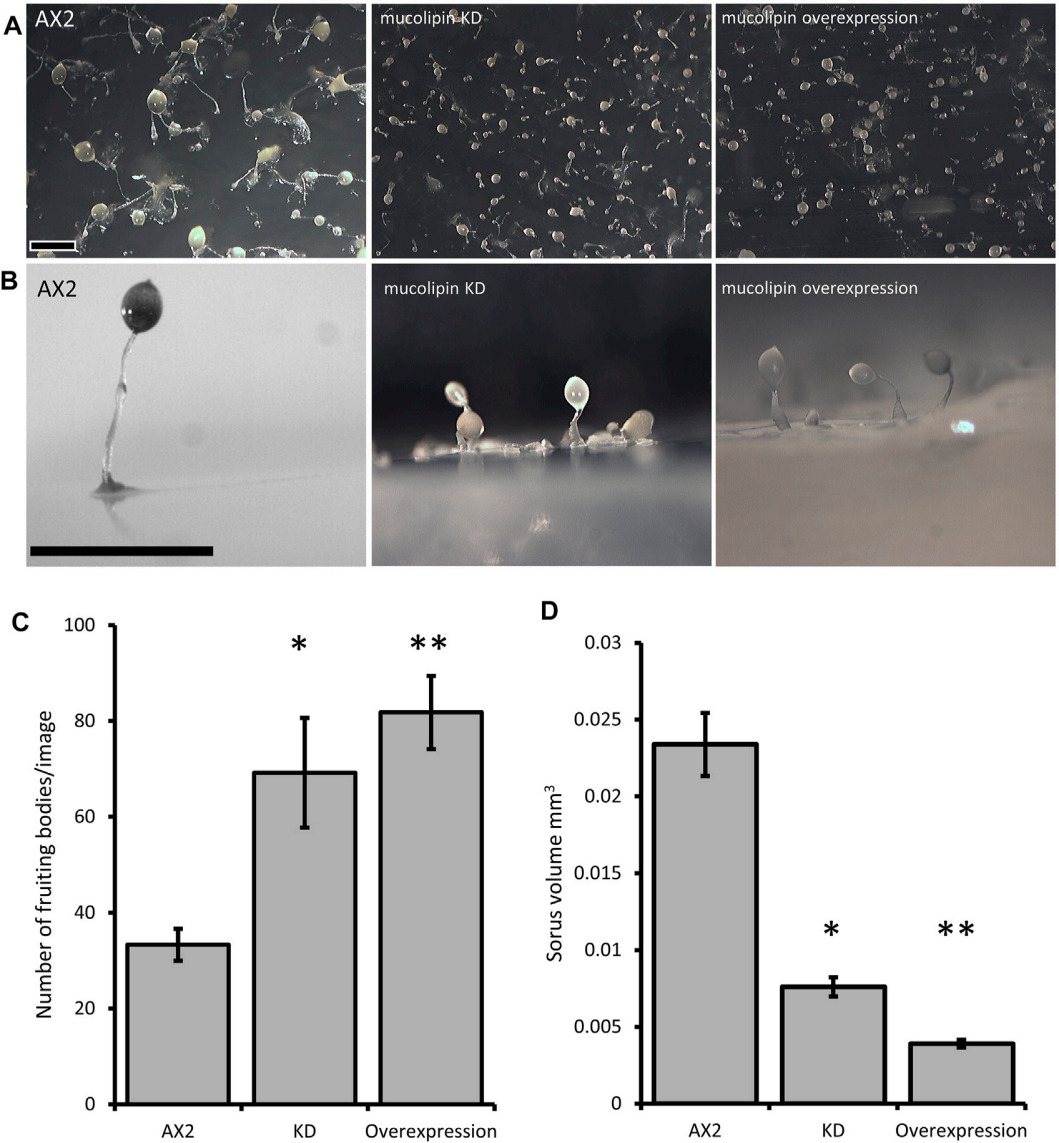
Mucolipin knockdown and overexpression strains form smaller more numerous fruiting bodies. **(A)** AX2 wild type, mucolipin KD (knockdown) strain HPF817 (copy number 450) and mucolipin overexpression strain HPF825 (copy number 600). Cells grown on lawns of *E. aerogenes* at 21°C for ∼24 h until fruiting bodies had formed which were photographed from above. **(B)** The same strains photographed from the side. Both increasing and decreasing mucolipin expression results in very small fruiting bodies compared to that of AX2. In high copy number strains the stalks and basal disks are thickened and enlarged. Scale bar = 1 mm. **(C)** The number of fruiting bodies were significantly increased in mucolipin knockdown and overexpression strains. Images were taken from above at the same magnification for all strains and the number of fruiting bodies in the same area of photograph were counted and the means calculated. (AX2: *n* = 3; KD: *n* = 5, overexpression: *n* = 5) (independent *t*-test *p = 0.0470 vs. AX2, **p = 0.00303 vs. AX2). **(D)** Sorus volume was smaller in the knockdown and overexpression strains than AX2. Sorus area was quantified from photographs of fruiting bodies from above using the ImageJ measurement tool and then converted to volume on the assumption that the sorus is approximately spherical using equation (V = 4/3*A/sqrt (A/pi) (A = area) and presented as mm^3^. For each strain 30–111 sori were measured. The sorus size was significantly reduced in knockdown (*p* = 2.402 × 10^–20^ vs. AX2) and overexpression strains (**p = 1.8998 × 10^–56^ vs. AX2). Sample sizes: AX2—N = 3 images, *n* = 97 sori; KD—N = 5 strains, *n* = 349 sori; overexpression—N = 5 strains, *n* = 298 sori) (independent *t*-test).

**FIGURE 9:**
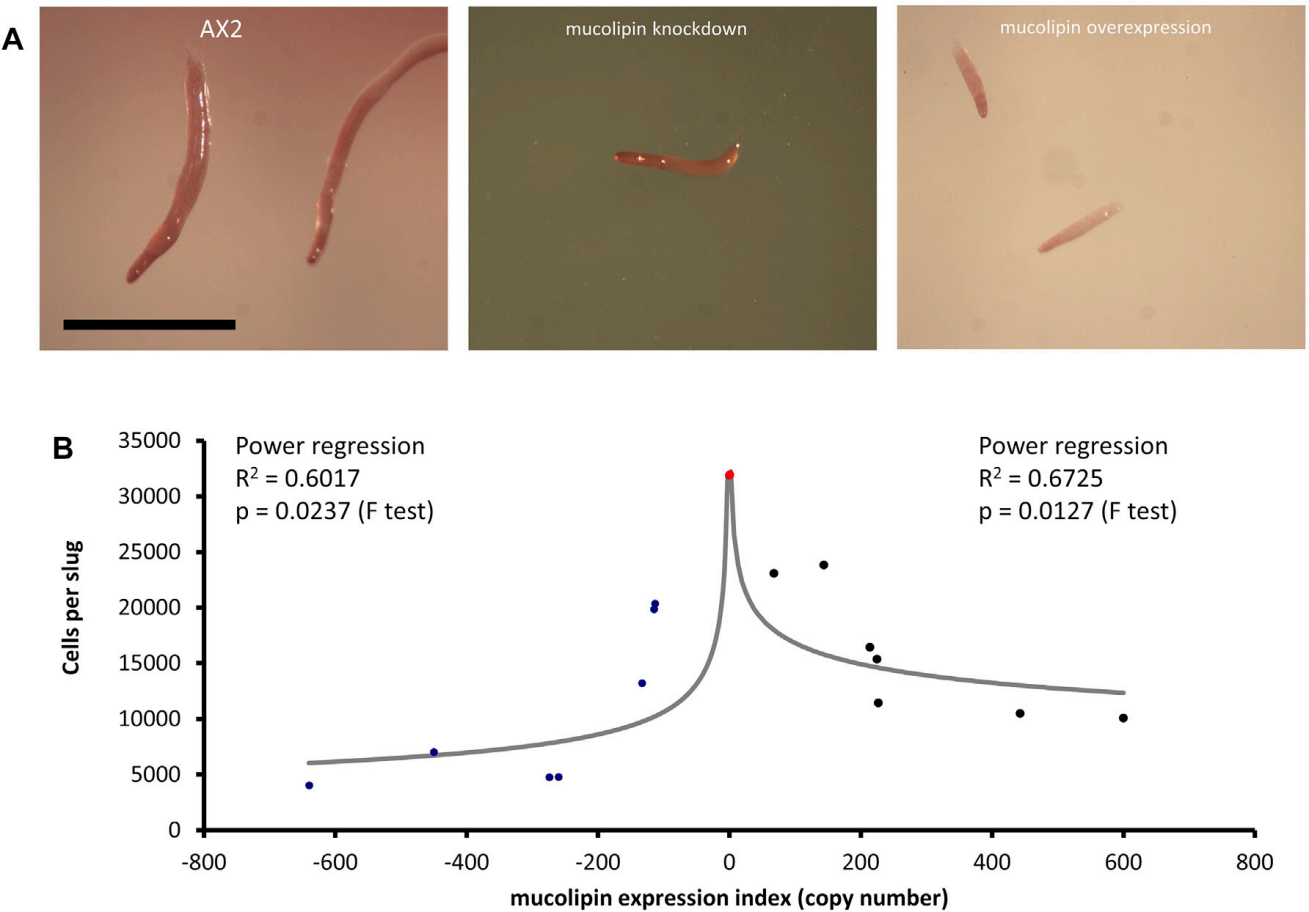
Slug sizes are smaller in mucolipin knockdown and overexpression strains and is copy number dependent. **(A)** Slugs formed after ∼16 h of development on water agar. Mutants formed smaller slugs than AX2 Scale bars = 1 mm. **(B)** Both increasing and decreasing mucolipin expression reduces aggregate size. The aggregate size of overexpression and knockdown strains was quantified by determining the number of cells per slug. The mean aggregate size for each transformant was determined by isolating single slugs and counting the number of cells in each slug using a haemocytometer (*n* = 30). The mean aggregate size was reduced in a copy number dependent manner, the regressions were significant indicated by the *p* values. Positive copy numbers represent overexpression strains and negative numbers knockdown strains, AX2 had a copy number of 0 as it contains neither plasmid (indicated as red circle).

Multicellular development in *Dictyostelium* is initiated by starvation upon which the cells undergo a developmental program leading to multicellular morphogenesis and culmination into mature fruiting bodies consisting of a spore droplet (sorus), stalk and basal disc. The stalk cells undergo autophagic cell death and so are nonviable. The developmental timing of expression of mucolipin suggests it is involved in chemotactic aggregation and progression through multicellular morphogenesis, likely through mucolipin-dependent calcium signalling, and its effects on the endocytic system. Calcium signalling plays an important role during development, from chemotactic aggregation to cell type differentiation. To investigate how expression levels of mucolipin affect multicellular morphogenesis in *Dictyostelium*, knockdown and overexpression strains were observed at the slug and fruiting body stage. Both knockdown and overexpression strains formed smaller fruiting bodies than AX2. In high copy number strains, the stalks and basal disks of the fruiting bodies appear thickened indicating increased stalk cell differentiation (Figures 8A,B), fruiting bodies were more numerous (Figures 8C) and formed smaller sori (Figures 8D). We then determined if the smaller fruiting bodies were the result of small aggregate and slug formation in the mutants. Indeed, both knockdown and overexpression strains developed into smaller slugs containing fewer cells compared to AX2 (Figure 9A). The aggregate size was quantified by determining the number of cells per slug, revealing a dramatic copy number-dependent reduction in slug size in both overexpression and knockdown strains (Figure 9B). This phenotype is also present in *cln2* knockdown mutants (Smith et al., 2019), and similarly *cln3* ^*−*^ and *cln5* ^*−*^ mutants have increased numbers of tipped mounds, fingers, slugs and fruiting bodies for the same density of cells (Huber et al., 2014; Huber et al., 2017; McLaren et al., 2021).

## 4 Discussion

In this study we have used the model organism *Dictyostelium* to investigate if mucolipin expression can affect global calcium signals during chemotactic calcium responses. Gross (2009) suggested that the *Dictyostelium* acidic stores, which includes the endolysosomal vesicles, the contractile vacuole system and the acidocalcisomes, can release Ca^2+^ and increase cytosolic Ca^2+^ during chemotaxis. A selection of calcium channels have been identified to be associated with the acidic stores including the P2X receptors of the contractile vacuole (Fountain et al., 2007), the two pore channel (TPC) (Wilczynska et al., 2005; Chang et al., 2020), and mucolipin. Given that mucolipin resides in late endosomes and other endocytic vesicles (Lima et al., 2012), we hypothesized that mucolipin could contribute to calcium release from these stores. We measured cytosolic Ca^2+^ responses to chemoattractants in mucolipin knockdown and overexpression transformants using the luminescence of the Ca^2+^-sensitive luminescent protein, apoaequorin, which we ectopically expressed in the same cells (Nebl and Fisher 1997). We found that overexpression of mucolipin significantly increased the magnitudes of the cytosolic Ca^2+^ responses to folic acid and cAMP. While we cannot determine if the increased calcium influx into the cytosol is the result of direct release of calcium from the endocytic compartments through mucolipin itself, our results do implicate mucolipin in cytosolic calcium signalling. Lima et al. (2012) provided evidence of mucolipin involvement in calcium homeostasis because knockout cells grew faster in calcium-depleted medium and had reduced lumenal calcium concentrations in the post-lysosomes, below 0.2 µM, much lower than the 1–3 µM of the wild type control. In other cell types TRPML1 has been shown to affect lysosomal pH (Soyombo et al., 2006; Miedel et al., 2008), therefore, knocking out mucolipin in *Dictyostelium* could affect the proton/Ca^2+^ exchange between the vesicle lumen and cytoplasm, thereby reducing steady state Ca^2+^ levels in the lumen as has been reported (Lima et al., 2012). This could in turn mean that overexpressing mucolipin may result in elevated resting lumenal Ca^2+^, again because of the disturbed pH gradient across the vesicle membrane. This, and the greater number of available channels could then facilitate the larger responses in our overexpressing strains. Lima et al. (2012) hypothesized that mucolipin may be responsible for transfer of calcium from the cytosol to the lumen where the vesicles meet high local cytosolic calcium concentrations. Our results suggest that, at least when overexpressed, mucolipin significantly contributes to cytosolic signals, thus could function as a calcium release channel. It is possible that the channel could work both ways depending on the particular cellular requirements.

As stated previously, in other cell types TRPML channels are known to regulate localised calcium signals, as well as global calcium waves via activation of ER and plasma membrane calcium flux (Kilpatrick et al., 2016). The same may be true in *Dictyostelium*—if mucolipin is capable of activating calcium release from ER and across the plasma membrane, then overexpressing the channel would enhance the cytosolic calcium responses as we observed in our experiments. It is also possible that overexpression of mucolipin results in expression at the plasma membrane, as this has been reported for TRPML1 expression in HEK293 cells (Kiselyov et al., 2005), or it may be translocated there during exocytosis as TRPML1 is in *Xenopus* oocytes (LaPlante et al., 2002). The *Dictyostelium* channels responsible for plasma membrane and ER calcium release are still not confirmed. Contenders are IplA, a channel related to the mammalian ER IP_3_ receptors (Taylor et al., 1999; Lusche et al., 2012) and pkd2 the *Dictyostelium* polycystin-2, another member of the TRP superfamily (Lima et al., 2014; Traynor and Kay, 2017). Both IplA and pkd2 have been localised to the plasma membrane (Lusche et al., 2012; Lima et al., 2014). However, IplA localises primarily to unidentified cytoplasmic inclusions, and it has not been determined whether this includes the ER (Lusche et al., 2012). Some studies have found that knockout of IplA abolishes calcium responses to chemoattractants (Traynor et al., 2000; Traynor and Kay, 2017), however another reported that the Ca^2+^response to high concentrations of cAMP is maintained but smaller (Schaloske et al., 2005). Calcium responses to cAMP and folic acid are retained in pkd2 knockout cells (Traynor and Kay, 2017). Therefore, clarification of which channels involved in ER and plasma membrane calcium signalling is still necessary. Future work to investigate if mucolipin contributes to calcium influx across the plasma membrane and release from the ER calcium could include measurement of the uptake of ^45^Ca^2+^ from the extracellular milieu, treatment of overexpression cells with thapsigargin to reduce ER calcium content (Lytton et al., 1991), and with EGTA to chelate extracellular calcium.

We also analysed cytosolic calcium signals in mucolipin knockdown strains, and were surprised to find that reducing mucolipin expression resulted in larger, rather than smaller Ca^2+^ responses. However, this occurred only in vegetative cells, the magnitudes of the calcium responses in differentiated cells stimulated with cAMP were not significantly different from controls (Figure 4). The enhanced Ca^2+^ responses observed in our vegetative mucolipin knockdown strains might be caused by a decrease in Ca^2+^ buffering capacity by the acidic stores. Lysosomes can shape calcium signals by tempering cytosolic calcium released from the ER (López-Sanjurjo et al., 2013). It has been reported that the ability of lysosomes to buffer Ca^2+^ in lysosomally diseased cells is greatly reduced, possibly because of the accumulation of undigested lysosomal cargo (Lloyd-Evans et al., 2008). Furthermore, buffering of cytosolic calcium by the mitochondria is defective in MLIV cells (Jennings et al., 2006). As calcium buffering is a mechanism involved in tempering calcium responses, loss of buffering capacity could actually result in enhanced global calcium signals. A similar mechanism has been demonstrated in *Dictyostelium* cells lacking the ER calcium binding proteins calnexin and calreticulin, as these strains had much larger calcium responses to chemoattractants than control cells (Wilczynska et al., 2005).

Since our mucolipin knockdown strains exhibited increased, as opposed to decreased, cytosolic Ca^2+^ signals during the vegetative phase of the lifecycle, it was surprising to find that when these strains were allowed to differentiate to aggregation-competence, their Ca^2+^ responses to cAMP were unaffected. Similarly, Chang et al. (2020) reported that in *Dictyostelium* TPC-null cells the magnitude of cAMP-mediated Ca^2+^ responses was comparable to that in wild type AX2 cells. Together these results suggest that mucolipin-dependent calcium signalling is not a major contributor to cytosolic calcium responses at this stage of development. Instead the role of mucolipin in the differentiated cells may be restricted to regulating local calcium signalling. Accordingly, the cAMP responses in the TPC null cells were slightly delayed in the time of onset, and this may be related to local calcium release from the acidic stores in “priming” ER calcium release (Chang et al., 2020), but this will require further investigation.

In previous work, the cellular phenotypes of growth, endocytosis and multicellular development were unaffected in mucolipin knockout cells created from the parental strain DH1-10 (Lima et al., 2012). Since our strains were made in the parental strain AX2, we wanted to also characterise these phenotypes in our strains. We found that surprisingly our strains did have abnormal phenotypes and many, but not all of these, presented similarly in the knockdown cells as overexpression cells. Furthermore, some of the phenotypes in our strains were similar to those reported in *Dictyostelium* NCL models.

Phagocytosis rates were increased in both mucolipin knockdown and overexpression cells. This may be directly related to the increased calcium signalling in these strains as evidence suggests that calcium is involved in phagocytosis (Muller-Taubenberger et al., 2001; Yuan et al., 2001; Fajardo et al., 2004; Pikzack et al., 2005). Pinocytosis rates were increased in knockdown strains, but unaffected in overexpression strains which suggests the defect is not directly caused by the abnormal calcium signalling. The involvement of Ca^2+^ in macropinocytosis is unclear. Extracellular Ca^2+^ is not essential (Williams and Kay, 2018), but both liberation of intracellular Ca^2+^ by caffeine treatment and inhibition of Ca^2+^ transport by La^3+^ treatment reduces macropinocytosis (Gonzalez et al., 1990). However, both caffeine and La^2+^ can affect other processes. Our results suggest different roles for mucolipin function in macropinocytosis and phagocytosis.

When measuring growth rates, we expected that the increase in phagocytosis rates would correlate with an increase in growth rates on bacterial lawns, however both knockdown and overexpression strains grew normally on *E. coli*. Furthermore, axenic growth rates were slightly reduced in knockdown strains, so again did not correlate with the increase in pinocytic uptake. This phenotype was also present in *cln2* knockdown mutants (Smith et al., 2019) and together these results may represent the presence of compensatory feedback pathways that upregulate rates of endocytosis as a response to nutrient deprivation caused by defective endolysosomal trafficking. This kind of reverse coupling of growth and endocytosis rates has also been observed in relation to other *Dictyostelium* lysosomal proteins. A *Dictyostelium* knockout of *alyA* (encoding the major lysozyme isoform) has 40% reduction in total lysozyme activity, exhibits slow growth on bacterial lawns but increased phagocytosis of fluorescently labelled yeast cells due to a compensatory pathway (Müller et al., 2005). Knocking down expression of lysosomal Tpp1 (tripeptidyl peptidase I encoded by *cln2/tpp1*) caused reductions in the growth rate in liquid medium, slower plaque expansion rates on bacterial lawns, but elevated rates of phagocytosis (Smith et al., 2019). These effects were mediated by reduced activity of the *Dictyostelium* TORC1 (homologue of the human mechanistic Target of Rapamycin Complex I) signalling pathway, being mimicked by rapamycin treatment (inhibitor of TORC1) and Rheb knockdown (upstream activator of TORC1), and rescued by Rheb overexpression (Smith et al., 2019). Other *Dictyostelium* NCL models have shown varying phenotypes in their growth and nutrient uptake phenotypes. Mutants lacking *cln3* and *cln5* displayed enhanced proliferation in HL5 medium, however pinocytic uptake was not significantly different from AX3, and the *cln3* ^*−*^ phenotype was rescued by overexpression of GFP-cln3 (Huber et al., 2014; McLaren et al., 2021). Combined these results indicate abnormalities in nutrient uptake and growth and the pathways that connect them in the different lysosomal disease models.

The defects in catabolism that we observed in our strains could also be caused by disruptions in mucolipin-mediated Ca^2+^-dependent vesicle fragmentation/fusion which is characteristic of MLIV and other MLIV models (Berman et al., 1974; Bargal and Bach, 1997; Lubensky et al., 1999). We observed that mucolipin knockdown and overexpression strains had increased fluorescence when stained with Lysosensor Blue. This may be linked to accumulation of Lysosensor Blue-stained vesicles as a result of Ca^2+^-dependent increases in homotypic or heterotypic vesicle fusion during the endosomal mixing stage and subsequent failure to progress through to exocytosis. Studies in other models reported that the defective membrane trafficking can be caused by both knockout and overexpression of TRPMLs causing accumulation of large hybrid late endosome-lysosomal compartments (Berman et al., 1974; Lubensky et al., 1999) as well as enlargement and clustering of endosomes (Fares and Greenwald, 2001; Kim et al., 2007; Martina et al., 2009; Vergarajauregui et al., 2009). In *Dictyostelium* DH1-10 mucolipin knockout cells, a significant increase in generation of post-lysosomes was reported, however as this was coupled with enhanced rate of post-lysosome fusion with the plasma membrane, and subsequent exocytosis, so that there was no measurable build-up of vesicles (Lima et al., 2012). We did not measure exocytosis rates in our strains, so further experiments could determine if exocytosis is blocked in our strains and account for the accumulation.

Alternatively, because Lysosensor Blue fluorescence increases as the pH becomes more acidic, the increased fluorescence in our knockdown and overexpression strains could also be caused by a decrease in the pH of the vesicles. Dysregulated lysosomal pH is common to lysosomal storage disease cells, however this is generally linked to increased pH as reported in MLIV fibroblasts type (Bach et al., 1999) and most NCLs (Holopainen et al., 2001). However, decreased lysosomal pH has been described in some MLIV fibroblasts (Soyombo et al., 2006). It is important to note that *Dictyostelium* cells lacking mucolipin exhibited no change in the pH of endosomal compartments (Lima et al., 2012).

A further consequence of dysfunctional catabolism, retention of unprocessed nutrients and decreased release of amino acids from the vesicles, could be that the cells are in a state of partial starvation and this would favour the initiation of aggregation. In support of this, both mucolipin knockdown and overexpression strains exhibited increased numbers of aggregation centers and accordingly formed slugs and fruiting bodies significantly smaller than those of AX2. This implicates mucolipin in activation of aggregation center formation and could be linked to calcium-dependent regulation of developmental processes (Sakamoto et al., 2003; Poloz and O’Day, 2012). The same phenotype is present in *IplA* ^*−*^ cells which form smaller mounds and fruiting bodies due to fragmented aggregation streams (Traynor et al., 2000; Schaloske et al., 2005). Similar phenotypes are also present in other *Dictyostelium* lysosomal disease models. Mutants with knocked down *cln2*/*tpp1* form smaller aggregates and fruiting bodies (also mediated by reduced TORC1 signalling, Smith et al., 2019), and *cln2*/*tpp1* ^*−*^ mutants progress faster through development (Phillips and Gomer, 2015). Cells lacking *cln3* and *cln5* exhibit precocious development, increased numbers of tipped mounds, fingers and slugs, similar to our mucolipin strains (Huber et al., 2014; Huber et al., 2017; McLaren et al., 2021). Interestingly, the precocious development in *cln3* ^*−*^ cells was rescued by chelation of calcium with EGTA. This implicates Cln3 in calcium regulation, likely due to its localisation at the contractile vacuole, a major calcium regulatory organelle (Malchow et al., 2006; Huber et al., 2014), and suggests calcium dysregulation may be common to lysosomal disease cells.

In addition to the increased numbers of aggregates formed in our knockdown and overexpression mutants, an increased proportion of the cells in the aggregates appear to be entering the autophagic cell death pathway. This was suggested by the proportionately thicker stalks and enlarged basal disks, a phenotype associated with increased autophagic cell death (Bokko et al., 2007). This is another phenotype common among *Dictyostelium* lysosomal disease mutants. It is also present in *tpp1* knockdown cells where it is mediated by reduced TORC1 signalling (Smith et al., 2019). Furthermore, enhanced autophagy has been reported in *tpp1* knockout cells (Phillips and Gomer, 2015) and *cln5* ^*−*^ cells display aberrant autophagy (McLaren et al., 2021). TPC knockout cells also accumulate autophagosomes (Chang et al., 2020) which further implicates calcium signalling through the acidic stores in autophagic processes. As autophagic cell death occurs late in the developmental cycle during stalk formation, it is possible that during autophagy, mucolipin together with other calcium channels in that location (TPC in particular) could play similar roles in generating a local Ca^2+^ cloud to facilitate fusion of the autophagosome and lysosome during formation of the stalk cell vacuole. Schaap et al. (1996) showed that a sustained elevation of resting cytoplasmic Ca^2+^ levels mediates late stalk gene (*ecmB*) induction by the morphogen DIF (Differentiation Inducing Factor) in *Dictyostelium.* Mucolipin could play a role in this process. In that case, as we have seen in the endolysosomal pathway, mucolipin overexpression and knockdown would both cause increased Ca^2+^ signals and subsequent disturbances in autophagolysosome formation, possibly explaining the stalk phenotype. An increase in cellular autophagic vacuoles is a characteristic associated with MLIV (Vergarajauregui et al., 2008) is observed both in mouse models (Curcio-Morelli et al., 2010), and *Drosophila* mucolipin knockout flies (Wong et al., 2012). The underlying nature of the autophagic defect may be related to disruption in signalling by the nutrient stress sensor mTORC1, a key regulator of autophagy. One study has shown that mTORC1 can directly phosphorylate TRPML1 to negatively regulate channel activity and decrease autophagy (Onyenwoke et al., 2015). To add further complexity, recent evidence has revealed that TRPML1 Ca^2+^ release can regulate mTORC1 autophagic pathways through a nutrient-sensitive negative feedback loop (Sun et al., 2018). Furthermore, TRPML1 is directly involved in the Ca^2+^/calmodulin dependent protein kinase β (CaMKKβ) activation of AMP-activated protein kinase (AMPK) (Scotto-Rosato et al., 2019). AMPK in turn inhibits TORC1 and thereby activates autophagy.

The nature of these complex signalling pathways still needs to be unravelled, therefore our study supports the view that *Dictyostelium* offers a tractable, simple model for MLIV cytopathology. The results suggest it would be valuable in future experiments to investigate further the signalling pathways involved in mucolipin Ca^2+^ signalling-dependent regulation of cellular growth and autophagy through *Dictyostelium* TORC1. This is of particular interest because the role of Ca^2+^ signalling in regulation of mTORC1 autophagy pathways is evident, but details are still unclear (Decuypere et al., 2011), as are mTORC1-TRPML signalling pathways. Therefore, it would be relevant to explore whether the growth and developmental defects can be rescued by genetically altering *Dictyostelium* TORC1 and *Dictyostelium* AMPK expression in mucolipin knockdown and overexpression backgrounds.

The results we have presented here highlight common phenotypes amongst *Dictyostelium* lysosomal disease models suggesting they may share common pathologies. We have also shown that both increasing and decreasing mucolipin expression levels can cause the same phenotypic outcome. Our results are similar to other studies where reports of both increased and decreased expression of mucolipin proteins can cause problems along the endocytic pathways. For example, when TRPML3 is knocked down there are defects in endosomal acidification and also increased homotypic endosomal fusion (Lelouvier and Puertollano, 2011), while when TRPML3 is overexpressed, endosomes become enlarged (Martina et al., 2009; Lelouvier and Puertollano, 2011). In *C. elegans*, both mucolipin knockout and some overexpression coelomocytes, exhibited the formation of large vacuoles (Fares and Geenwald, 2001). Similarly, overexpression of TRPML1 results in accumulation of enlarged endosomes containing both early (Hrs) and late (CD63) endocytic markers (Vergarajauregui et al., 2009) and induces an aberrant distribution of these compartments within the cell (Manzoni et al., 2004). These combined reports show that the endocytic pathway is clearly impacted by disturbances in both directions in the expression levels or activities of mucolipin proteins and the resultant alterations in Ca^2+^ signalling.

## Data Availability

Raw data will be made available by contacting the corresponding authors, without reservation.
