# Supplementary material for: The Dictyostelium Model for Mucolipidosis Type IV
Source: Front Cell Dev Biol. 2022 Apr 13;10:741967. doi: 10.3389/fcell.2022.741967 (PMC9043695; doi:10.3389/fcell.2022.741967)
Supplement: Supplementary file 1 [file DataSheet1.PDF]

Supplementary Information

Supplementary Method 1. Mass spectrometry identification of proteins in Bis-Tris-Plus gel slices. Cells were grown in HL5 medium and 1 × 10^6 cells harvested and lysed in 100 µl 1 × Bolt LDS sample buffer (Thermo Fisher Scientific) with a protease inhibitor 1 cocktail (Complete-EDTA free, Roche) on ice from 30 min then centrifuged at 1200 rpm for 2 mins. 10 µl of lysate was with 1% Bolt sample reducing agent (Thermo Fisher Scientific) was incubated at 70°C for 10 min then loaded onto a Bolt™ 4 to 12%, Bis-Tris-Plus, 1.0 mm, Protein Gel, 12-well (Invitrogen) with the Broad Multi Color Pre-Stained Protein Standard (Genscript) and subject to electrophoresis. The gel containing protein was stained with 0.25 % Coomassie brilliant (Bio Rad) blue for 1 h and subsequently destained (5% MeOH, 7.5% Acetic Acid) to visualise the protein bands. Protein bands at ~120 and ~95 kDa were excised using a sterile scalpel and placed in 100 µl of sterile milliQH_2O. The proteins contained in the bands were identified by mass spectrometry at the La Trobe University-Proteomics and Metabolomics Platform according to the following protocol.

Excised SDS-PAGE gel bands were destained in 50% acetonitrile, 50 mM ammonium bicarbonate (ABC) until clear of Coomassie dye before dehydration in 100 % acetonitrile. Protein in the gel band was reduced with 2 mM tris-2-carboxyethyl-phosphine (TCEP) in 20 mM ABC (60 min incubation), followed by exchange to iodoacetamide (40 mM in ABC for 45 min, in darkness). The gel band was then washed in 50% acetonitrile, 20 mM ABC for 30 min, dehydrated with 100 % acetonitrile, before rehydration with sequencing grade trypsin (125 ng, Promega) in 100 uL of 20 mM ABC. Trypsin digestion was completed overnight at 37 °C. Tryptic peptides were recovered as the supernatant and the gel band was then subjected to repeated dehydration in 85% acetonitrile, 0.5% Trifluoracetic acid (TFA) to extract the remaining peptides in the gel band. Recovered peptides were concentrated on a speedvac and resuspended in 15 uL of 5 % acetonitrile, 0.5% TFA. Samples were briefly centrifuged to pellet any debris before transfer to mass-spec vials for LC-MS analysis of the peptides.

Tryptic peptides were separated on a Thermo Ultimate 3000 RSLCnano UHPLC system and Thermo Q-Exactive HF Orbitrap mass-spectrometer (Thermo-Fisher Scientific, Waltham, MA, USA). Peptides (5 µL) were loaded onto a PepMap C18 5 µm 0.5 cm trapping cartridge (Thermo-Fisher Scientific, Waltham, MA, USA) and washed at 10 µL/min for 6 min (Buffer C: 0.1% (v/v) trifluoracetic acid, 2% (v/v) ACN) before switching the pre-column in line with the analytical column held at 55 °C (nanoEase M/Z Peptide BEH C18 Column, 1.7 µm, 130 Å and 75 µm ID × 25 cm, Waters). The separation of peptides was performed at 250 nL/min using a linear ACN gradient of buffer A (0.1% (v/v) formic acid, 2% (v/v) ACN) and buffer B (0.1% (v/v) formic acid, 80% (v/v) ACN), starting at 12% buffer B to 30% over 54 min, then rising to 50% B over 10 min followed by 95% B in 6 min. The column was then cleaned for 5 min at 95% B and then afterward a 13 min short equilibration step completed at 1% B. Blanks were run between sample injections.

Data were collected with Orbitrap HCD parameters including Data Dependent Acquisition (DDA) using as MS scan range and CID MS/MS spectra collected. Dynamic exclusion parameters were set as follows: exclude isotope on, exclude after n = 1 times, duration 60 s, charge state 2-7, cycle time was set to 3 seconds, and used the peptide monoisotopic peak determination mode. Other instrument parameters were: MS1 scan at 60,000 resolution, m/z 350–1500, AGC target 3e6, injection time max 30 ms. MS2 scans were at 60,000 resolution, m/z 200–2000, AGC target 1e5, injection time max 110 ms. The top seven ions were fragmented per cycle. The isolation window of the quadrupole for the precursor was 1.4 m/z. Lock mass was set using m/s 445.12003 for internal mass calibration.

Raw files were searched using PEAKS studio XPRO (v 10.6). Search was performed against the Dictybase reference proteome. The protein database was supplemented with sequences from the Common Repository of Adventitious Proteins to account for typical lab contaminants (https://www.thegpm.org/crap). Fixed modification of carbamidomethyl cysteine, and variable modifications of oxidation of methionine, acetylation of protein n-terminus, carbamylation of lysine and protein n-terminus were used in the search. The parent ion mass error tolerance was 10.0ppm and the fragment mass error tolerance was 0.05 Da. Up to three missed trypsin cleavages were permitted and the search false discovery rate was set to a maximum of 1%.

Supplementary Figure 1.

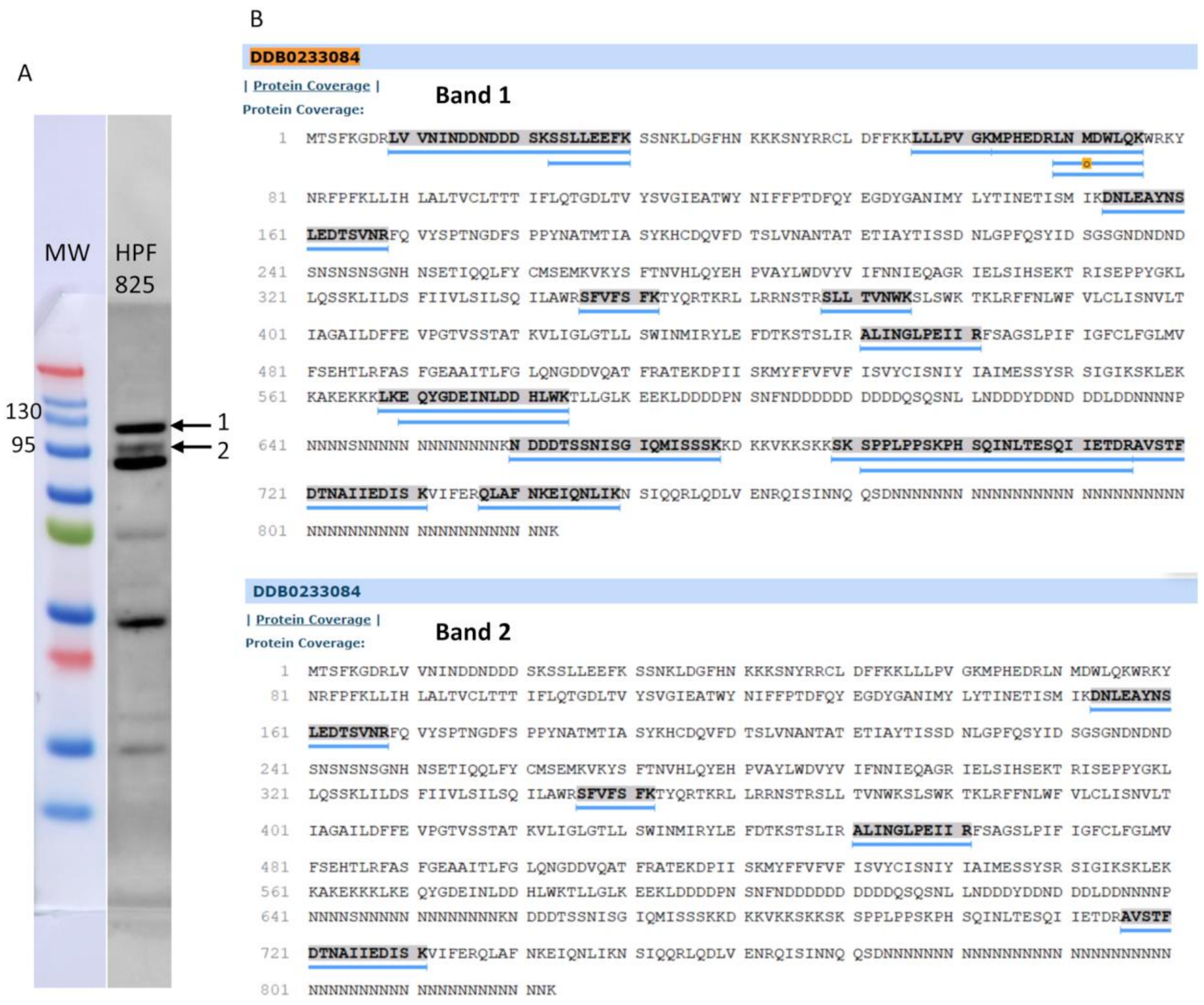

Supplementary Figure 1. Identification of mucolipin bands in western blots. A crude protein lysate from a mucolipin overexpressing strain, HPF825, was run in an SDS-PAGE gel and western blotting was used to visualize the bands that reacted with the antimucolipin antibody. Multiple bands were observed. Bands 1 and 2 were present in lysates from overexpression strains, including HPF825 (Panel A) but were not visible in western blots of crude lysates from wild type, knockdown and knockout strains (Figure 3, main text). B. These bands were excised and the proteins therein were identified by LC-MS (Supplementary Method 1). The 120 and 95 kDa bands (Bands 1 and 2) both contained mucolipin as indicated by the identified peptides (Panel B).
